# Sustainable Syntheses of (−)-Jerantinines A & E and Structural Characterisation of the Jerantinine-Tubulin Complex at the Colchicine Binding Site

**DOI:** 10.1038/s41598-018-28880-2

**Published:** 2018-07-13

**Authors:** Christopher J. Smedley, Paul A. Stanley, Mohannad E. Qazzaz, Andrea E. Prota, Natacha Olieric, Hilary Collins, Harry Eastman, Andrew S. Barrow, Kuan-Hon Lim, Toh-Seok Kam, Brian J. Smith, Hendrika M. Duivenvoorden, Belinda S. Parker, Tracey D. Bradshaw, Michel O. Steinmetz, John E. Moses

**Affiliations:** 1 La Trobe Institute for Molecular Science, La Trobe University, Melbourne, VIC 3086 Australia; 2 School of Pharmacy, University of Nottingham, University Park, Nottingham, NG7 2RD UK; 3 Laboratory of Biomolecular Research, Division of Biology and Chemistry, Paul Scherrer Institute, CH-5232 Villigen PSI, Switzerland; 4 University of Basel, Biozentrum, CH-4056 Basel, Switzerland; 5 School of Pharmacy, University of Nottingham Malaysia Campus, Jalan Broga, 43500 Semenyih, Selangor Malaysia; 6 Department of Chemistry, Faculty of Science, University of Malaya, 50603 Kuala Lumpur, Malaysia

## Abstract

The jerantinine family of *Aspidosperma* indole alkaloids from *Tabernaemontana corymbosa* are potent microtubule-targeting agents with broad spectrum anticancer activity. The natural supply of these precious metabolites has been significantly disrupted due to the inclusion of *T. corymbosa* on the endangered list of threatened species by the International Union for Conservation of Nature. This report describes the asymmetric syntheses of (−)-jerantinines A and E from sustainably sourced (−)-tabersonine, using a straight-forward and robust biomimetic approach. Biological investigations of synthetic (−)-jerantinine A, along with molecular modelling and X-ray crystallography studies of the tubulin—(−)-jerantinine B acetate complex, advocate an anticancer mode of action of the jerantinines operating *via* microtubule disruption resulting from binding at the colchicine site. This work lays the foundation for accessing useful quantities of enantiomerically pure jerantinine alkaloids for future development.

## Introduction

Natural products have made an enormous contribution in the treatment of cancer, with over half of the current anticancer drugs in clinical use being natural products or natural product derivatives^1–3^. Sometimes, however, there are disadvantages when using natural products as lead structures in drug discovery. Typically isolated from source in only minute quantities, the challenge of accessing useful quantities of these precious metabolites can present significant bottlenecks for development. This is particularly conspicuous when the natural supply has been exhausted and, in such instances, synthesis remains the only viable option. However, secondary metabolites are often incredibly challenging targets with unprecedented molecular connectivity and structural complexity^4^, and even when successful, total synthesis does not always result in adequate quantities of material for developmental studies^5,6^.

The tubulin binding^7,8^ *Vinca* indole alkaloids, including vincristine and vinblastine are classic examples of structurally complex natural products that are among the foremost drugs used in cancer chemotherapy^1–3,9^. However, owing to their structural complexity, the *de novo* synthesis of these important drugs remains a significant challenge^10,11^, and clinical supplies of vincristine and vinblastine primarily rely upon natural sources^10–15^. Another major limitation to the continued use of the *Vinca* alkaloids is the emergence of drug resistance, derived primarily from the overexpression of phosphoglycoprotein (Pgp) efflux pump, that is responsible for transporting many of the major drugs out of the cell^16,17^. Accordingly, the discovery and synthesis of bioactive alkaloids that overcome these resistance mechanisms is of high priority for anticancer drug development.

In 2008, several new *Aspidosperma* indole alkaloids, (−)-jerantinines A–G, were isolated by Kam and co-workers, from a leaf extract of the Malayan *Tabernaemontana corymbosa* Roxb. ex Wall^18^. The jerantinines displayed pronounced *in vitro* cytotoxicity toward drug-sensitive as well as vincristine-resistant (VJ300) KB cells (IC_50_ < 1 μg/mL)^18^, which is uncommon among simple *Aspidosperma* alkaloids. Studies have shown that both (−)-jerantinines A (**1**) and E (**3**) are microtubule-destabilising agents (MDAs)^19,20^, whereas **1** also induces tumour-specific cell death through modulation of splicing factor 3b subunit 1 (SF3B1)^21^.

The antiproliferative and pro-apoptotic activities of the jerantinines *via* mechanisms involving perturbation of validated antitumor targets warrant further investigation as potential chemotherapeutic agents. However, the inclusion of *T. corymbosa* on the endangered list of threatened species by the International Union for Conservation of Nature (IUCN)^22^, limits the source of these natural compounds, and a practical synthetic route is urgently required.

To date, there have been only three reported synthetic studies towards the jerantinine alkaloids. Waser *et al*. reported the first synthesis of jerantinine E (**3**), which involved a key homo-Nazarov cyclisation of an aminocyclopropane to access four of the five rings, leading to racemic (±)-(**3**) in 17-steps and 16% overall yield^20^. In 2017, Magauer and co-workers described a convergent synthesis to the ABC ring system of jerantinine E (**3**) that employed a *β*-C–H enone bromination followed by a palladium-catalysed amination and oxidative indole formation to yield a tricyclic core^23^.

Very recently, Jiang and co-workers completed the first total syntheses of (−)-jerantinines A (**1**), C (**2**) and E (**3**) in 12–13 steps^24^. The key steps of this route included a stereoselective intramolecular inverse-electron demand [4 + 2] cycloaddition, an α,β-unsaturated ketone indolisation and a Pd/C-catalysed cascade reaction.

Continuing our work on the biomimetic synthesis of natural products^25–27^, we report here the asymmetric and sustainable semi-syntheses of both (−)-jerantinine A (**1**) and (−)-jerantinine E (**3**), and provide supporting biological and structural data to further advocate a mechanism of action of the jerantinines *via* microtubule disruption.

**Figure 1:**
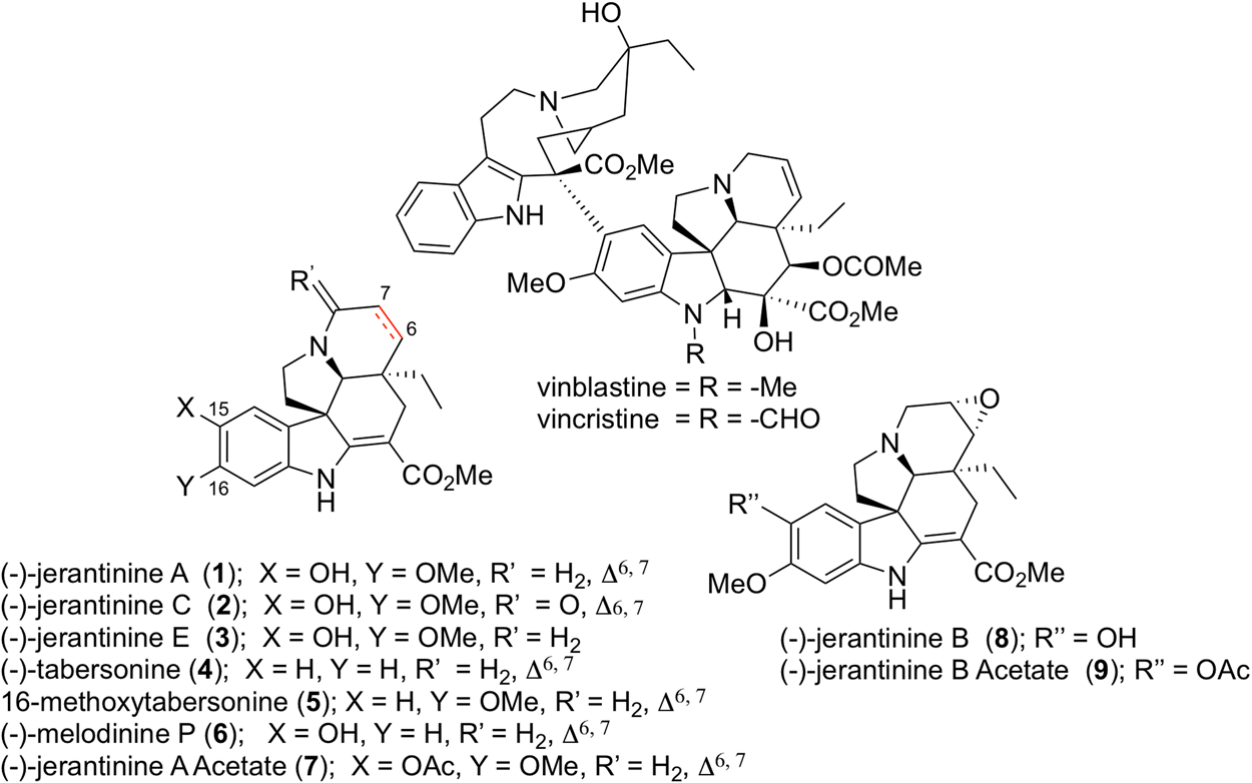
A selection of *Vinca* and *Aspidosperma* alkaloids.

**Figure 2:**
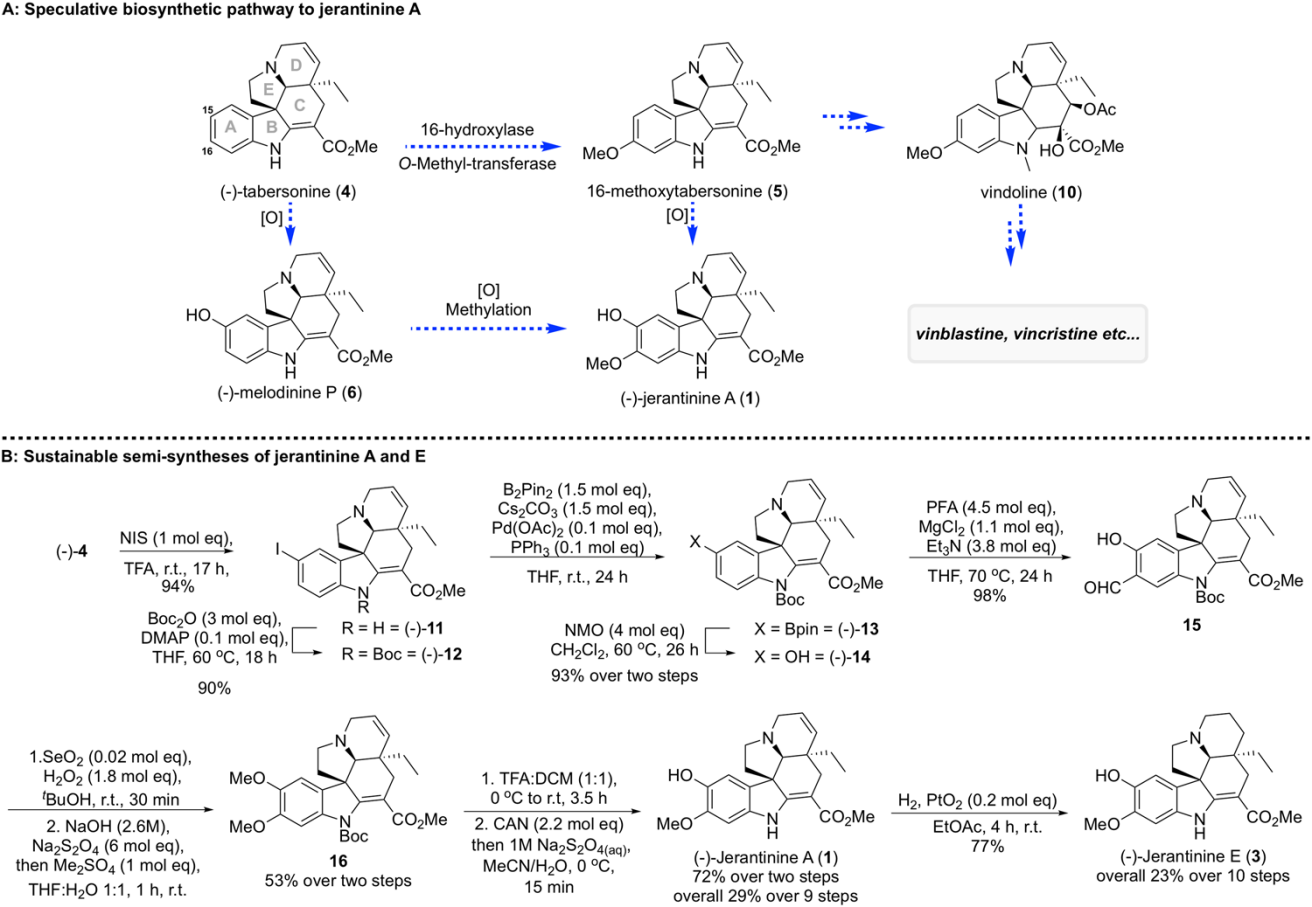
(**A**) Speculative biosynthetic pathway of jerantinine A and related vinca alkaloids (**1**) (**B**) Sustainable biomimetic semi-synthesis of (−)-jerantinine A (**1**) and (−)-jerantinine E (**3**), beginning from the proposed biogenetic precursor, (−)-tabersonine (**4**).

The indole alkaloid (−)-tabersonine (**4**) was proposed as a plausible precursor to the jerantinines — diverging from the vindoline biosynthetic pathway^28,29^, a selective C-15^30^ A-ring oxidation of 16-methoxytabersonine (**5**) would give **1** directly^31,32^. Alternatively, the C16 *ortho*-hydroxylation and subsequent methylation of the metabolite melodinine P (**6**) (from *Melodinus suaveolens*) would directly give **1** (Figs 1 and 2)^33^.

Thus, inspired by Nature’s efficiency in creating vast numbers of complex molecules from a single precursor, and the opportunity to exploit a readily available and sustainable natural feedstock, we elected to explore tabersonine (**4**) as a platform for synthetic elaboration into (−)-jerantinine A (**1**)^34–36^.

Studies commenced with the multi-gram scale extraction of **4** from the ground seeds of *Voacanga Africana*^37,38^, giving consistent yields of up to 1.4% by mass recovery. The reaction of **4** with *N-*iodosuccinimide in TFA at room temperature gave exclusively the 15-iodotabersonine (**11**) in 94% yield^39^. We anticipated that a Miyaura borylation followed by oxidation would conveniently install the required C-15 hydroxyl^40–42^, but upon experimentation we found that protection of the indoline nitrogen was first required to prevent unwanted side reactions. Gratifyingly, the *N*-Boc-iodotabersonine **12** reacted smoothly to give the boronate ester **13**, which upon exposure to NMO yielded hydroxytabersonine **14** in 93% yield over 2 steps^41^. The next challenge was to install the critical C-16 hydroxyl, and we elected here to investigate a classical *ortho*-formylation followed by Dakin oxidation sequence; given the sterically more encumbered C-14 ring position, we were confident that this could be achieved with good regiochemical control. This proved to be robust and convenient; thus, upon treatment of the Boc-protected **14** with paraformaldehyde in the presence of MgCl_2_ and Et_3_N, the C-16-aldehyde **15** was isolated in 98% yield^43^. Exposing **15** to a solution of hydrogen peroxide with a trace amount of SeO_2_^44^ in *tert*-butanol gave a complex mixture, and therefore an operationally convenient one-pot procedure was developed; aldehyde **15** was fully oxidised to the quinone, back-reduced with sodium dithionite and the catechol trapped by methylation to give the dimethoxy derivative **16** in 53% yield. Finally, sequential *N*-Boc deprotection and selective demethylation under Waser’s conditions^20^, furnished (−)-jerantinine A (**1**) in 72% yield, and 29% overall yield from (−)-tabersonine (**4**). Synthetic jerantinine A (**1**) was shown to be identical to natural jerantinine A through comparison of their ^1^H and ^13^C NMR spectroscopic data (Table S1). The conversion of **1** into (−)-jerantinine E (**3**) was accomplished upon selective reduction of the Δ^6,7^ double bond through H_2_ over PtO_2_ in 77% (23% overall yield, Scheme 1).

**Figure 3:**
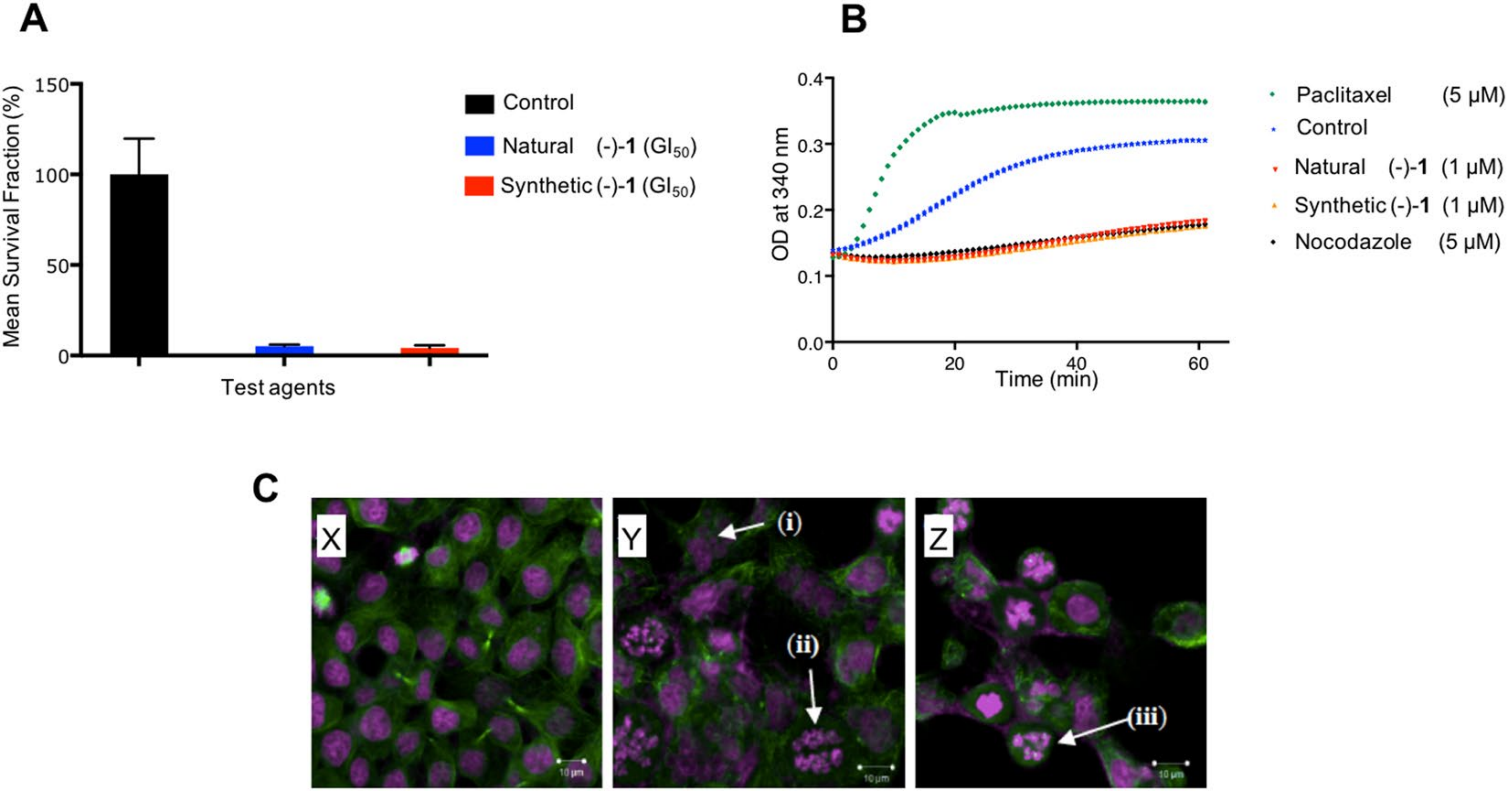
(**A**) Effect of natural and synthetic **1** on HCT-116 colony formation at GI_50_ (see Table 1). Mean survival fraction (%) of treated cells as a percentage of the control population for HCT-116. Cells were seeded (400 per well) and allowed 24 h to attach before being challenged with jerantinine A (24 h). The number of colonies forming after 8 d incubation was determined. Mean (±S.E.M.) values are given. Data were generated from ≥3 separate trials; n = 2 per trial; (**B**) *In vitro* tubulin polymerisation in the presence of vehicle control, paclitaxel (5 μM; positive control), nocodazole (5 μM; negative control) or **1** (1 µM); (**C**) Immunofluorescent and DRAQ5 staining of x) untreated MCF-7 cells and y-z) cells exposed to synthetic **1** (1 × GI_50_ = 0.81 μM; 24 h), showing the effect on microtubules (green) and the cellular DNA (purple). Synthetic (−)-jerantinine A (**1**) caused multinucleation (i), nuclear fragmentation (ii) and formation of multipolar spindles (iii).

With significant amounts of synthetic **1** and synthetic intermediates *en route* to **1**, we next explored the cytotoxicity of these molecules (See SI, Table S-2). The human-derived MCF-7 breast and HCT116 colorectal carcinoma cell lines were chosen, since they are representative of two of the most prevalent types of cancer. *In vitro* antitumour activity of synthetic **1** was indistinguishable from the natural compound; for example, in MTT tests GI_50_ values <1 μM against colorectal and mammary human-derived carcinoma cell lines were observed for both samples of **1** (Table 1) - dose-response profiles are shown in supplementary data (Fig. S-1). Furthermore, both the synthetic and natural **1** dramatically and significantly inhibited colony formation of HCT116 cells when treated at the GI_50_ concentration (Fig. 3A). Cell cycle analyses demonstrated that at 0.8 µM, both natural and synthetic **1** profoundly perturbed the cell cycle, causing stark arrest at G2/M phase (See SI, Fig. S-1). Collectively, these data advocate that mitotic microtubule assembly has been disrupted. Indeed, at 1 μM naturally sourced and synthetic (−)-**1** both abolished tubulin polymerisation *in vitro* as profoundly as the 5 μM nocodazole control (Fig. 3B). MCF-7 cells were treated for 24 h with 0.8 µM **1**, stained (DRAQ5 and monoclonal anti α-tubulin Ab) and the images viewed by confocal microscopy. As shown in Fig. 3C, many characteristic features of the action of a microtubule disrupting agent (MDA) were observed, including formation of multipolar spindles, misaligned chromosomes, multinucleation (aneuploidy) and nuclear fragmentation^45,46^.

**Table 1:** GI_50_ values of natural product and synthetic jerantinine A (**1**) and colchicine against human-derived colorectal (HCT116) and mammary (MCF-7) carcinoma cell lines.

	mean GI_50_ ± SD (μM)		
	Natural 1	Synthetic 1	Colchicine
HCT116	0.76 ± 0.13	0.82 ± 0.07	0.03 ± 0.01
MCF-7	0.85 ± 0.09	0.81 ± 0.07	

Data calculated from n ≥ 3 trials; n = 8 per concentration point per trial.

**Figure 4:**
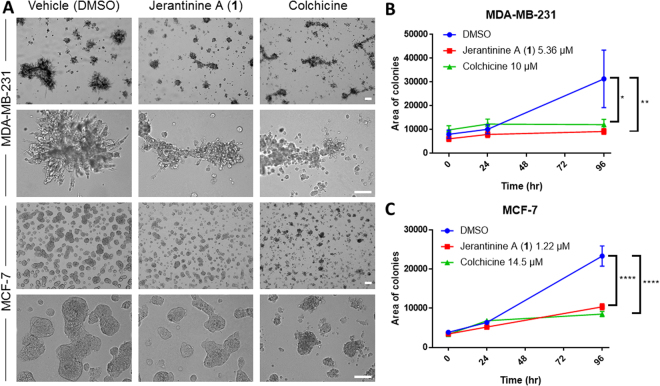
(**A**) MDA-MB-231 and MCF-7 breast cancer cells grown in 3D culture with vehicle (DMSO), jerantinine A (**1**) (5.36 µM or 1.22 µM‚ respectively) or colchicine (10 µM or 14.5 µM‚ respectively). Images from 96 h post agent exposure. Scale bar represents 200 µm. (**B**) Quantification of the area of the MDA-MB-231 3D colonies over a 96 h period post agent addition. Mean (±S.E.M.) values are given. (**C**) Quantification of the area of the MCF-7 3D colonies over a 96 h period post agent addition. Mean (±S.E.M.) values are given. Data were generated from ≥2 separate trials; n = 2 per trial; *p < 0.05, **p < 0.01, ****p < 0.0001.

Following these results, we tested the impact of jerantinine A (**1**) and colchicine on breast cancer cell growth in 3D culture (Fig. 4). After 96 h of exposure to either agent, the cell density of both cell lines (measured as area) was decreased compared to control (Fig. 4B and C). Jerantinine A (**1**) decreased cell density of the MDA-MB-231 to a greater extent than colchicine following 96 h of exposure (Fig. 4B). The invasive nature of the MDA-MB-231 triple negative breast cancer cell line was also decreased by treatment with jerantinine A (**1**) or colchicine.

**Figure 5:**
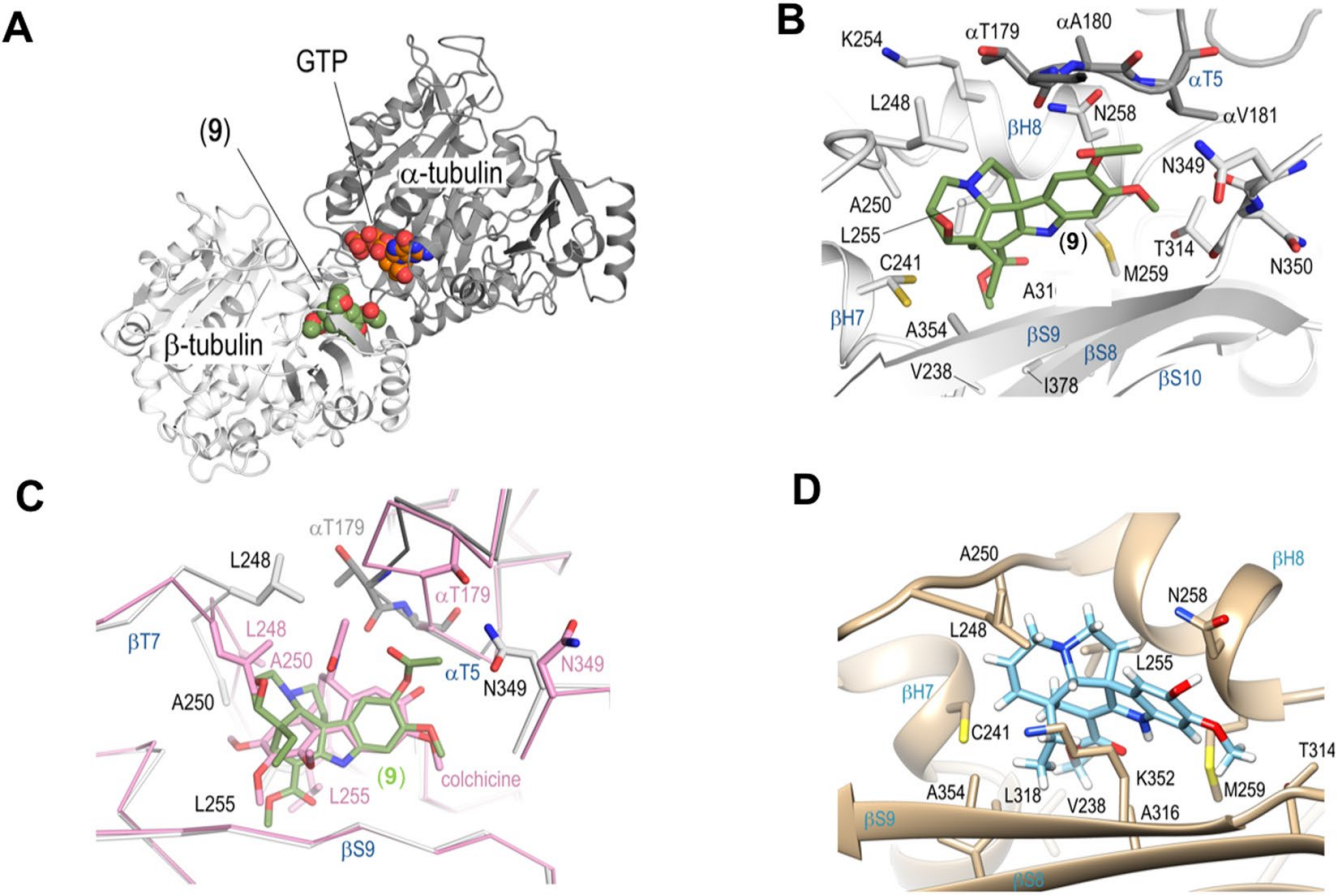
Crystal structure of the tubulin-jerantinine B acetate (**9**) complex. (**A**) Overall view of the T_2_R-TTL-jerantinine B acetate complex structure. The α- and β-tubulin chains are in dark and light grey ribbon representation, respectively. The tubulin-bound **9** and GTP molecules are represented as green and orange spheres, respectively; (**B**) Close-up view of the interactions observed between **9** (green) and tubulin (grey). Interacting residues of tubulin are shown in stick representation and are labelled. Oxygen and nitrogen atoms are coloured red and blue, respectively; carbon atoms are in green **9** or grey (tubulin). Secondary structural elements of tubulin are labelled in blue. For simplicity, only α-tubulin residues are indicated with an α; (**C**) Superimposition of the tubulin-**9** (green/grey) and the tubulin-colchicine (pink; PDB ID 4O2B; rmsd of 0.204 Å over 351 Cα-atoms) complex structures. The structures were superimposed onto their β1-tubulin chains; (**D**) Molecular docking of (−)-jerantinine A (**1**) in the colchicine binding pocket of tubulin; this pocket is formed by residues in the αT5-loop on α-tubulin, and βH7, βH8, βS8, βS9 and the βT7-loop on β-tubulin. For additional experimental details see the Supporting Information.

Soaking experiments with a selection of the jerantinine metabolites and derivatives into a crystal formed by a protein complex composed of two αβ-tubulin heterodimers, the stathmin-like protein RB3 and tubulin tyrosine ligase (T_2_R-TTL)^47,48^, yielded the tubulin-jerantinine B acetate (**9**) structure at 2.4 Å resolution (Fig. 5A; Table S-3). The overall structure of the two tubulin dimers in the T_2_R-TTL-**9** complex superimposed well with the one obtained in the absence of a ligand^49^ (Fig. S-3A; rmsd_overall_ of 0.48 Å over 1814 Cα-atoms; rmsd_chain B_ 0.24 Å over 351 Cα-atoms). This result suggests that binding of the ligand does not affect the gross conformation of tubulin. Thus, the complex structure reveals that **9** binds to the colchicine site of tubulin that is shaped by residues of strands βS8 and βS9, loop βT7, and helices βH7 and βH8 of β-tubulin, and loop αT5 of α-tubulin (Fig. 5B).

Superimposition of the tubulin-**9** structure onto the tubulin-colchicine structure (rmsd of 0.204 Å over 351 Cα-atoms, chain B)^49^ revealed a perfect overlap of the jerantinine A-ring of **9** with the C-ring of colchicine and two major conformational changes in the binding site of **9** (Fig. 5C). In the jerantinine structure, Thr179 on the αT5 loop is in the flipped-in conformation and forms a hydrophobic contact to Leu248 on the βT7 loop. In the colchicine structure Thr179 is in the flipped-out conformation to avoid clashes with the acetamide moiety. Moreover, jerantinine-binding induces a rearrangement of Leu248 on the βT7 loop to prevent clashes with the epoxide moiety on the D-ring of **9** (Fig. 4C), and of Ala250 and Leu255 to fill the space that is otherwise occupied by the A-ring of colchicine.

Tubulin dimers in microtubules assume a “straight” structure; in contrast, free tubulin is characterised by a “curved” conformation^50,51^. The jerantinine B acetate (**9**) prevents the curved-to-straight structural transition by sterically hindering the βT7 loop adopting its conformation characteristic of the straight tubulin state (Fig. S-3B), a mechanism that is similar to other colchicine-site ligands^49,51–53^.

Subsequent molecular docking studies revealed a distinct preference of jerantinines A (**1**) and E (**3**) for the colchicine pocket (Fig. 5D, Table S-4)^54^. Binding energies to either the vinblastine or taxol sites were approximately 10 and 15 kJ mol^−1^, respectively, lower than the colchicine site. Jerantinine B acetate (**9**) was calculated as the tightest binding ligand of the family, and was predicted to bind ~5 kJ mol^−1^ more tightly than colchicine. The structure of jerantinine B acetate closely matches the experimentally determined structure – rmsd of 0.68 Å. The predicted binding orientation of all the jerantinine derivatives, in the colchicine binding site, mirror that of jerantinine B acetate (**9**).

In conclusion, we have described the asymmetric synthesis of the *Aspidosperma* alkaloid (−)-jerantinine A (**1**) in 9 steps and 29% overall yield, using a sustainable semi-synthetic approach. The selective reduction of (−)-**1** enabled the asymmetric synthesis of the corresponding (−)-jerantinine E (**3**). Our biomimetic approach began from the natural building block and plausible biosynthetic precursor (−)-tabersonine (**4**), that is readily available in large quantities from a sustainable plant-based source (*Voacanga Africana*). The synthesis enabled us to investigate the biological profile of (−)-jerantinine A (**1**), showing that activity of synthetic **1** is indistinguishable from that of the natural material against human derived breast (MCF-7) and colon carcinoma (HCT116) cell lines. A 3D culture assay showed that jerantinine A (**1**) decreased the cell density of two breast cancer cell lines, in particular demonstrating greater effectiveness than colchicine against MDA-MB-231 cells. Investigations into the mode of action suggested that **1** acts *via* disruption of the microtubule network, as indicated by its potent inhibitory activity displayed in tubulin polymerisation *in vitro*. This particular mode of action was further corroborated by solving of the crystal structure of the tubulin—jerantinine B acetate complex, revealing binding to the colchicine site and prevention of the “curved-to-straight” tubulin conformational transition that is necessary to enable microtubule formation.

## Electronic supplementary material

Supplementary Information
