# Supplementary material for: Sustainable Syntheses of (−)-Jerantinines A & E and Structural Characterisation of the Jerantinine-Tubulin Complex at the Colchicine Binding Site
Source: Sci Rep. 2018 Jul 13;8:10617. doi: 10.1038/s41598-018-28880-2 (PMC6045569; doi:10.1038/s41598-018-28880-2)

Sustainable Syntheses of (-)-Jerantinines A & E and Structural Characterisation of the Jerantinine-Tubulin Complex at the Colchicine Binding Site

Christopher J. Smedley[1], Paul A. Stanley[2], Mohannad E. Qazzaz[2], Andrea E. Prota[3], Natacha Olieric[3], Hilary Collins[2], Harry Eastman[2], Andrew S. Barrow[1], Kuan-Hon Lim[5], Toh-Seok Kam[6], Brian J. Smith[1], Hendrika M. Duivenvoorden[1], Belinda S. Parker[1],Tracey D. Bradshaw[2], Michel O. Steinmetz[3,4] and John E. Moses*[1]

[1] La Trobe Institute for Molecular Science, La Trobe University, Melbourne VIC 3086. E-mail: J.Moses@latrobe.edu.au. [2] School of Pharmacy, University of Nottingham, University Park, Nottingham NG7 2RD, UK [3] Laboratory of Biomolecular Research, Division of Biology and Chemistry, Paul Scherrer Institute CH-5232, Villigen PSI, Switzerland. [4] University of Basel, Biozentraum, CH-4056 Basel, Switzerland. [5] School of Pharmacy, University of Nottingham Malaysia Campus, Jalan Broga, 43500 Semenyih, Selangor, Malaysia. [6] Department of Chemistry, Faculty of Science, University of Malaya, 50603 Kuala Lumpur, Malaysia.

Contents

1. Experimental 4

1.1 General Procedure 4

1.2 Experimental procedures 5

1.2.1 Extraction of (-)-tabersonine (4)[1] 5

1.2.2 (-)-15-Iodo-tabersonine (11)[2] 6

1.2.3 (-)-*N*-Boc-15-iodo-tabersonine (12) 7

1.2.4 *N*-Boc-tabersonine-15-Boronic acid pinacol ester (13) 9

1.2.5 *N*-Boc-15-hydroxy-tabersonine (14) 10

1.2.6 *N*-Boc-15-hydroxy-16-formyl-tabersonine (15) 11

1.2.7 *N*-Boc-15,16-dimethoxy-tabersonine (16) 12

1.2.8 Jerantinine A (1)[3] 14

1.2.9 Jerantinine E (3)[3] 17

1.2.10 Jerantinine A acetate (7)[3] 18

2. Biological Data 19

2.1 Methods and Materials 19

2.1.1 Agent stocks 19

2.1.2 Cell culture 19

2.1.3 MTT assay 19

2.1.4 Clonogenic assay 19

2.1.5 Cell cycle analysis 19

2.1.6 Tubulin polymerisation assay 20

2.1.7 Confocal Microscopy 20

2.1.8 3D culture 20

2.2 Results. 21

2.2.1 Anti-cancer properties of the intermediates in the synthesis of jerantinine A, as determined by MTT assays 21

2.2.2 Cell cycle analysis 23

2.2.3 Confocal Microscopy 24

2.3 Analysis 25

2.3.1 MTT assay 25

2.3.2 Cell cycle analysis 25

2.3.3 Jerantinine A and jerantinine A acetate inhibit tubulin polymerisation 25

2.3.4 Confocal Microscopy 26

2.3.5 Discussion 26

3. X-ray crystallography data 27

3.1 Crystallisation, data collection and structure determination 27

4. Modelling of Binding Studies 31

4.1 Methods 31

4.2 Results 31

5. References 33

6. Spectroscopic Data 35

1. Experimental

1.1 General Procedure

Unless otherwise stated, all reactions were performed in flame-dried glassware under an atmosphere of argon with magnetic stirring. Tetrahydrofuran (THF) was freshly distilled over sodium wire and benzophenone; Dichloromethane (CH_2Cl_2) was freshly distilled over CaH_2 under a nitrogen atmosphere. Triethylamine (Et_3N) was distilled from CaH_2 and stored over 4 Å molecular sieves. All other reagents and solvents were obtained from commercial sources and used without further purification. The progress of reactions were monitored using thin layer chromatography (TLC) and visualised using UV light and stained using a basic $KMnO_4$ (potassium permanganate) solution. Flash column chromatography was performed using Merck silica gel 60 as the stationary phase. Petrol refers to petroleum spirit (b.p. 40-60 °C).

NMR spectra (^1H and ^{13}C) were recorded on Bruker Ascend™ 400 (400 MHz) and Ultrashield™ 500 PLUS (500 MHz) spectrometers, in $CDCl_3$. Infra-red spectra were obtained using a Bruker Tensor 27 FT-IR spectrophotometer as a solution in $CHCl_3$, with the peaks recorded as v_{max} /cm^{-1}. HRMS were obtained using an Agilent 6530 accurate-mass Q-TOF LC/MS in electrospray ionisation (ESI) or electron ionisation (EI) mode. Melting point data were collected using a Stuart SMP3. Optical rotations were recorded using ADP440 polarimeter.

1.2 Experimental procedures

1.2.1 Extraction of (-)-tabersonine (4)[1]

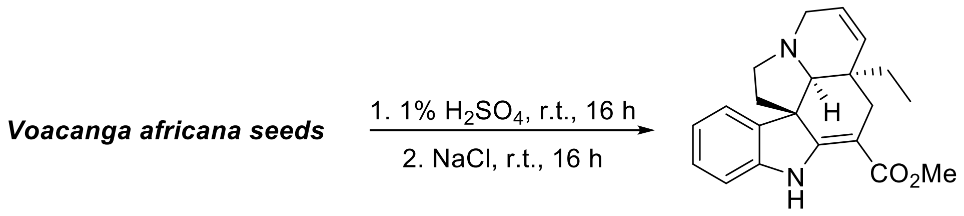

Scheme S-1. *Extraction of (-)-tabersonine.*

Voacanga africana seeds (200 g) were ground into a fine powder and suspended in an aqueous solution of 1% H_2SO_4 (2.0 L). The resulting mixture was then stirred at room temperature for 16 h. The acidic extract was then filtered and NaCl (200 g) added to the filtrate, and stirred for 16 h. $CHCl_3$ (1.5 L) was then added and the biphasic solution vigorously stirred for 3 h. After settling, the aqueous layer was removed and discarded and the remaining organic emulsion filtered through Celite®. The resulting solution was then separated and the organic fraction dried over anhydrous $MgSO_4$ and filtered. The solvent was then removed under reduced pressure and the resulting material dissolved in $CHCl_3$ (50 mL), followed by the addition of 30% aqueous ammonia (30 mL). The organic layer was separated and the aqueous phase extracted with $CHCl_3$ (3 x 50 mL). The combined organic fractions were then dried over anhydrous $MgSO_4$, filtered and the solvent removed under reduced pressure. The crude material obtained was purified by flash column chromatography (SiO_2, 0-10% EtOAc in petroleum ether 40-60 °C) to give (-)-tabersonine (**4**) (2.8 g, 1.4% by mass) as a white crystalline solid. m.p.: 78 °C; $[\alpha]_D$ –250.12 (*c* 0.10, $CHCl_3$).

IR ν_{max}/cm^{-1}: 3367, 3023, 2960, 1670, 1604, 1131.

^{1}H NMR (400 MHz, $CDCl_3$) δ_H = 9.00 (s, 1H), 7.24 (d, *J* = 7.3 Hz, 1H), 7.15 (td, *J* = 7.7, 1.2 Hz, 1H), 6.88 (td, *J* = 7.4, 1.2 Hz, 1H), 6.84 - 6.81 (m, 1H), 5.82 - 5.78 (m, 1H), 5.74 - 5.70 (m, 1H), 3.78 (s, 3H), 3.50 - 3.44 (m, 1H), 3.23 - 3.16 (m, 1H), 3.08 - 3.03 (m, 1H), 2.76 - 2.67 (m, 2H), 2.56 (dd, *J* = 15.1, 1.9 Hz, 1H), 2.45 (d, *J* = 15.1 Hz, 1H), 2.08 (td, *J* = 11.3, 6.5 Hz, 1H), 1.81 (ddd, *J* = 11.7, 4.7, 1.0 Hz, 1H), 1.08 - 0.96 (m, 1H), 0.92 - 0.82 (m, 1H), 0.65 (t, *J* = 7.4 Hz, 3H).

^{13}C NMR (100 MHz, $CDCl_3$) δ_C = 169.2, 166.9, 143.3, 138.2, 133.2, 127.8, 125.0, 121.6, 120.7, 109.4, 92.3, 70.2, 55.3, 51.1, 51.1, 50.7, 44.6, 41.5, 28.6, 27.1, 7.6.

HRMS (ESI$^+$): calculated for $C_{21}H_{25}N_2O_2$ $[M+H]^+$: m/z = 337.1916, m/z found = 337.1908.

1.2.2 (-)-15-Iodo-tabersonine (11)[2]

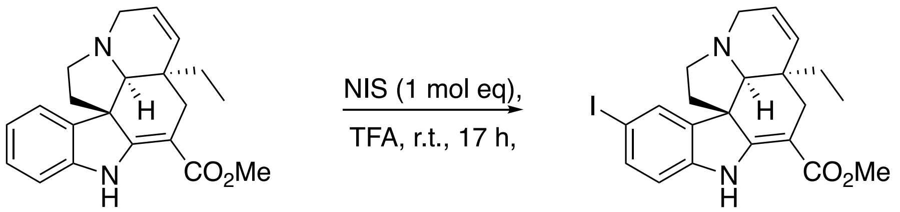

Scheme S-2: *Iodination of tabersonine.*

To a stirred solution of tabersonine (2.93 g, 8.71 mmol) in TFA (120 mL) was added NIS (1.96 g, 8.71 mmol) in 4 portions over 30 min. The reaction was allowed to stir for 17 h. The reaction mixture was then added slowly into an aqueous solution of NaOH (2 M, 1 L) at 0 °C and extracted with CH_2Cl_2 (4 x 50 mL). The combined organic fractions were then dried over anhydrous $MgSO_4$, filtered and the solvent removed under reduced pressure. The crude material was dissolved in pentane (250 mL), filtered and the solvent removed

under reduced pressure to give the title compound (**11**) as a white amorphous solid (3.79 g, 94%). m.p.: 85 °C; $[\alpha]_D$ −119 (*c* 0.10, $CHCl_3$).

IR ν_{max}/cm^{-1}: 3359, 3022, 2959, 1671, 1131.

^{1}H NMR (400 MHz, $CDCl_3$) δ_H = 8.99 (s, 1H), 7.51 - 7.49 (m, 1H), 7.44 (dd, *J* = 8.2, 1.7 Hz, 1H), 6.61 (d, *J* = 8.1 Hz, 1H), 5.81 - 5.76 (m, 1H), 5.73 - 5.68 (m, 1H), 3.77 (s, 3H), 3.45 (ddd, *J* = 15.9, 4.7, 1.4 Hz, 1H), 3.24 - 3.17 (m, 1H), 3.08 - 3.01 (m, 1H), 2.69 (ddd, *J* = 10.8, 8.5, 4.8 Hz, 1H), 2.63 (s, 1H), 2.55 (dd, *J* = 15.1, 1.8, 1H), 2.41 (d, *J* = 15.1, 1H), 2.06 (td, *J* = 11.2, 6.5 Hz, 1H), 1.82 (ddd, *J* = 11.7, 4.9, 1.4 Hz, 1H), 1.04 - 0.93 (m, 1H), 0.92 - 0.80 (m, 1H), 0.65 (t, *J* = 7.4 Hz, 3H).

^{13}C NMR (101 MHz, $CDCl_3$) δ_C = 169.1, 165.6, 143.1, 140.9, 136.6, 132.9, 130.5, 125.0, 111.5, 93.0, 82.6, 70.2, 55.2, 51.3, 51.1, 50.5, 44.6, 41.2, 28.6, 27.2, 7.6.

HRMS (ESI^+): calculated for $C_{21}H_{23}IN_2O_2$ $[M+H]^+$: m/z = 463.0882, m/z found = 463.0861.

1.2.3 (-)-*N*-Boc-15-iodo-tabersonine (12)

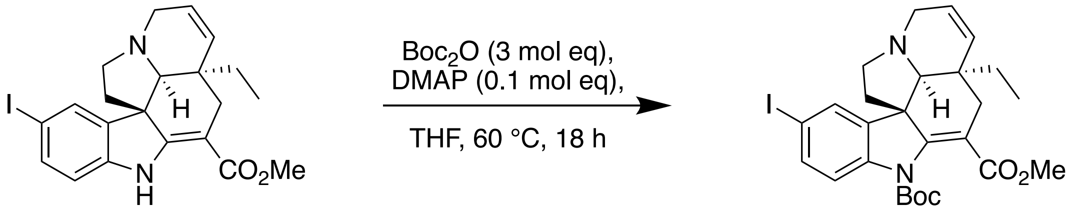

Scheme S-3. *Boc protection of (-)-15-iodo-tabersonine.*

To a stirred solution of (-)-15-iodo-tabersonine (11.9 g, 25.7 mmol) in anhydrous THF (250 mL) was added Boc_2O (8.20 mL, 77.1 mmol) and DMAP (313 mg, 2.57 mmol). The resulting mixture was heated at 60 °C for 18 h. The solvent was then removed under reduced pressure and the residue dissolved in CH_2Cl_2 (200 mL), followed by washing with water (3 x 50 mL). The organic fraction was dried over anhydrous $MgSO_4$ and filtered. The solvent was removed under reduced pressure to yield the crude product, which was

subsequently purified by flash column chromatography (SiO_2, 10% EtOAc in petroleum ether 40-60 °C) to give the title compound (**12**) as a white solid (13.1 g, 90%). m.p.: 83 °C; $[\alpha]_D$ −44.32 (*c* 0.10, $CHCl_3$).

IR ν_{max}/cm^{-1}: 2933, 2777, 1714, 1147.

^{1}H NMR (400 MHz, $CDCl_3$) δ_H = 7.57 - 7.52 (m, 1H), 7.48 - 7.46 (m, 1H), 7.45 - 7.42 (m, 1H), 5.83 - 5.77 (m, 1H), 5.68 - 5.62 (m, 1H), 3.76 (s, 3H), 3.49 (dd, *J* = 15.8, 4.9 Hz, 1H), 3.10 (d, *J* = 15.9 Hz, 1H), 3.04 (t, *J* = 7.7 Hz, 1H), 2.69 (d, *J* = 15.2 Hz, 1H), 2.57 (s, 1H), 2.50 (td, *J* = 10.6, 8.7, 5.2 Hz, 1H), 2.30 (dd, *J* = 15.2, 1.9 Hz, 1H), 2.15 (td, *J* = 11.8, 6.7 Hz, 1H), 1.79 (dd, *J* = 11.9, 5.0 Hz, 1H), 1.53 (s, 9H), 1.14 - 1.04 (m, 1H), 1.03 - 0.92 (m, 1H) 0.64 (t, *J* = 7.5 Hz, 3H).

^{13}C NMR (100 MHz, $CDCl_3$) δ_C = 168.0, 150.9, 149.3, 141.1, 140.7, 136.5, 132.5, 129.9, 125.3, 118.2, 112.1, 86.7, 82.8, 68.2, 52.7, 51.8, 51.5, 51.0, 43.1, 41.5, 31.2, 28.3, 26.5, 7.7.

HRMS (ESI$^+$): calculated for $C_{26}H_{32}IN_2O_4$ $[M+H]^+$: m/z = 563.1407, m/z Found = 563.1425.

1.2.4 *N*-Boc-tabersonine-15-Boronic acid pinacol ester (13)

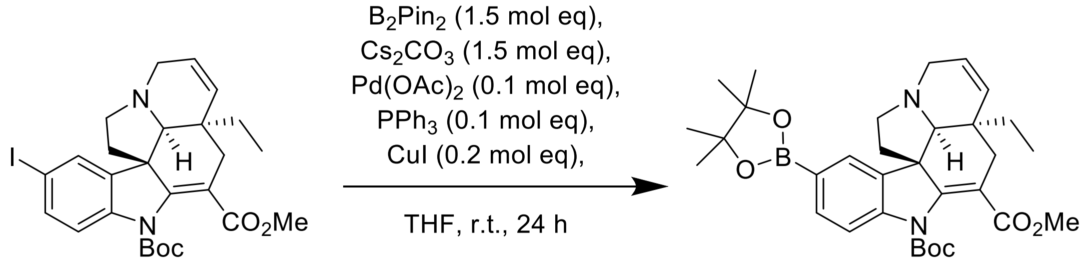

Scheme S-4. *Miyaura borylation of (-)-N-boc-15-iodo-tabersonine.*

To a stirred solution of *N*-boc-15-iodo-tabersonine (5.00 g, 8.80 mmol) in THF (250 mL) was added Cs_2CO_3 (4.30 g, 13.2 mmol), B_2Pin_2 (3.35 g, 13.2 mmol), CuI (335 mg, 1.76 mmol), PPh_3 (209 mg, 0.88 mmol) and $Pd(OAc)_2$ (197 mg, 0.88 mmol). The reaction mixture was stirred for 24 h. The reaction mixture was filtered through Celite® and then concentrated under reduced pressure to yield a brown solid. The resultant product was then used without further purification. A small sample was purified by flash column chromatography (SiO_2, 20% EtOAc in petroleum ether 40-60 °C) for analytical and biological purposes. m.p.: 94 °C; $[\alpha]_D$ –16.62 (*c* 0.10, $CHCl_3$).

IR ν_{max}/cm^{-1}: 2974, 2932, 1720, 1140.

^{1}H NMR (400 MHz, $CDCl_3$) δ_H 7.75 - 7.71 (m, 1H), 7.68 - 7.64 (m, 1H), 7.56 (s, 1H), 5.82 - 5.76 (m, 1H), 5.69 - 5.63 (m, 1H), 3.76 (s, 3H), 3.53 -3.45 (m, 1H), 3.17 (d, *J* = 15.9 Hz, 1H), 3.04 - 2.97 (m, 1H), 2.76 - 2.67 (m, 2H), 2.59 (ddd, *J* = 11.8, 8.6, 5.1 Hz, 1H), 2.34 - 2.26 (m, 1H), 2.14 (td, *J* = 11.8, 6.7 Hz, 1H), 1.76 (dd, *J* = 11.7, 4.9 Hz, 1H), 1.54 (s, 9H), 1.35 (s, 12H), 1.14 - 1.04 (m, 1H), 0.99 - 0.89 (m, 1H), 0.61 (t, *J* = 7.5 Hz, 3H).

^{13}C NMR (100 MHz, $CDCl_3$) δ_C = 168.2, 151.1, 150.3, 143.4, 138.0, 135.3, 132.7, 126.9, 125.2, 115.3, 111.6, 83.9, 82.5, 68.0, 52.6, 51.7, 51.5, 51.0, 43.2, 41.6, 31.0, 28.3, 26.4, 25.0, 25.0, 7.7.

HRMS (ESI^+): calculated for $C_{32}H_{44}BN_2O_6$ $[M+H]^+$: m/z = 563.3292, m/z Found = 563.3312.

1.2.5 *N*-Boc-15-hydroxy-tabersonine (14)

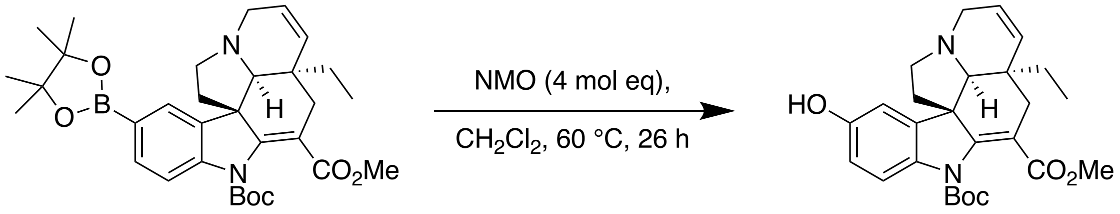

Scheme S-5. *Oxidation of N-boc-tabersonine-15-boronic acid pinacol ester.*

To a stirred solution of crude *N*-boc-tabersonine-15-Boronic acid pinacol ester (assumed 8.80 mmol) in CH_2Cl_2 (125 mL) was added NMO hydrate (4.75 g, 35.2 mmol) in 2 portions over 2 h. The reaction was refluxed at 60 °C for 24 h. The solvent was removed under reduced pressure and the crude product was purified by flash column chromatography (SiO_2, 30% EtOAc in petroleum ether 40-60 °C) to yield a white solid (**14**) (3.70 g, 93% yield over 2 steps) m.p.: 128 °C; $[\alpha]_D$ –30.33 (*c* 0.10, $CHCl_3$).

IR ν_{max}/cm^{-1}: 3347, 3025, 2767, 1720, 1683, 1112.

^{1}H NMR (400 MHz, $CDCl_3$) δ_H = 7.49 (d, *J* = 8.5 Hz, 1H), 6.74 (d, *J* = 2.6 Hz, 1H), 6.66 (dd, *J* = 8.6, 2.6 Hz, 1H), 5.83 - 5.76 (m, 1H), 5.67 - 5.61 (m, 1H), 4.99 (br. s, 1H), 3.76 (s, 3H), 3.48 (dd, *J* = 15.7, 4.7, 1H), 3.09 - 2.99 (m, 2H), 2.70 (d, *J* = 15.1 Hz, 1H), 2.57 (s, 1H), 2.53 - 2.42 (m, 1H), 2.28 (dd, *J* = 15.1, 1.8 Hz, 1H), 2.14 (td, *J* = 11.7, 6.7 Hz, 1H), 1.79 (dd, *J* = 11.8, 5.0 Hz, 1H), 1.54 (s, 9H), 1.16 - 1.04 (m, 1H), 1.03 - 0.93 (m, 1H), 0.63 (t, *J* = 7.4 Hz, 3H).

^{13}**C NMR** (100 MHz, $CDCl_3$) δ_C = 168.4, 152.2, 151.4, 150.1, 140.2, 134.3, 132.8, 125.3, 117.1, 113.6, 111.9, 109.1, 82.2, 68.2, 52.8, 51.8, 51.6, 51.1, 43.0, 41.6, 31.4, 28.4, 26.6, 7.8.

HRMS (ESI^+): calculated for $C_{26}H_{34}N_2O_5$ $[M+H]^+$: m/z = 453.2389, m/z Found = 453.2367.

1.2.6 *N*-Boc-15-hydroxy-16-formyl-tabersonine (15)

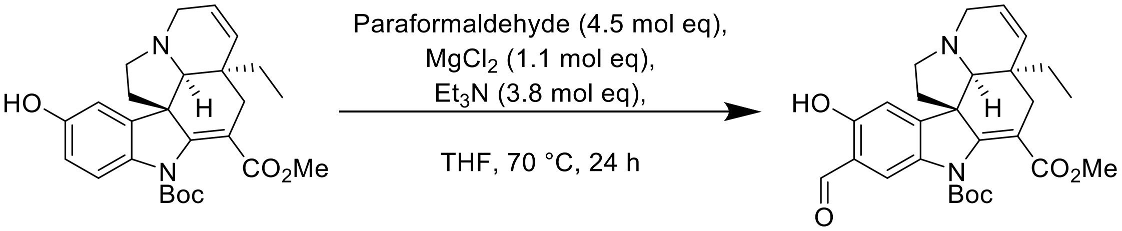

Scheme S-6. *Formylation of N-boc-15-hydroxy-tabersonine.*

To a stirred solution of *N*-boc-15-hydroxy-tabersonine (100 mg, 0.22 mmol) in THF (1 mL) was added $MgCl_2$ (23 mg, 0.25 mmol), Et_3N (114 μL, 0.85 mmol) and paraformaldehyde (30 mg, 1.00 mmol), and was then refluxed at 70 °C under an atmosphere of argon. After 24 h, the reaction mixture was poured into 1 M HCl (20 mL), neutralised with sat. $NaHCO_{3\,(aq)}$ and extracted with CH_2Cl_2 (3 x 10 mL). The organic fractions were combined and further washed with water (2 x 10 mL). The organic phase was dried over $MgSO_4$, filtered and the solvent removed under reduced pressure. The crude product was then purified by flash column chromatography (SiO_2, 40% EtOAc in petroleum ether 40-60 °C) to yield a yellow solid (**15**) (102 mg, 98% yield). m.p.: 190 °C; $[\alpha]_D$ -48.48 (*c* 0.10, $CHCl_3$).

IR ν_{max}/cm^{-1}: 2963, 2930, 1711, 1651, 1298, 1144.

^{1}**H NMR** (400 MHz, $CDCl_3$), δ_H = 11.28 (s, 1H), 9.84 (s, 1H), 7.85 (s, 1H), 6.87 (s, 1H), 5.84 - 5.78 (m, 1H), 5.66 - 5.61 (m, 1H), 3.77 (s, 3H), 3.50 (dd, *J* = 15.9, 4.9 Hz, 1H), 3.11 - 3.02 (m, 2H), 2.71 (d, *J* = 15.2 Hz, 1H), 2.61 (s, 1H), 2.54 - 2.44 (m, 1H), 2.30

(dd, J = 15.2, 1.8 Hz, 1H), 2.17 (td, J = 11.7, 6.6 Hz, 1H), 1.80 (dd, J = 12.0, 5.0 Hz, 1H), 1.54 (s, 9H), 1.12 - 0.80 (m, 2H), 0.63 (t, J = 7.4 Hz, 3H).

^{13}C NMR (101 MHz, $CDCl_3$) δ_C = 196.1, 167.8, 159.6, 151.2, 148.8, 148.7, 133.6, 132.4, 125.3, 119.4, 118.8, 112.6, 110.7, 82.8, 68.1, 53.3, 51.8, 51.6, 51.2, 43.1, 41.4, 31.3, 28.3, 26.5, 7.8.

HRMS (ESI$^+$): calculated for $C_{27}H_{33}N_2O_6$ $[M+H]^+$: m/z = 481.2339, m/z Found = 481.2392.

1.2.7 *N*-Boc-15,16-dimethoxy-tabersonine (16)

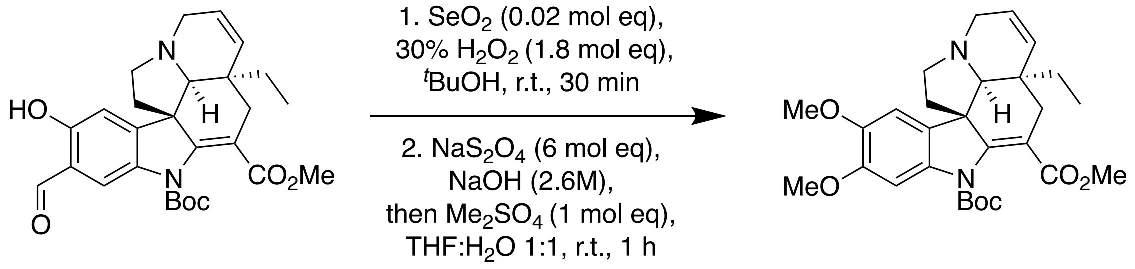

Scheme S-7. *Baeyer-Villiger reaction of N-boc-15-hydroxy-16-formyl-tabersonine, followed by demethylation of the intermediate.*

To a stirred solution of *N*-boc-15-hydroxy-16-formyl-tabersonine (50 mg, 0.10 mmol) in ^{t}BuOH (1 mL) was added SeO_2 (0.2 mg, 0.002 mmol) and 30% H_2O_2 (18 μL, 0.18 mmol) and the mixture stirred at room temperature for 30 min. The reaction mixture was then poured into saturated aqueous NaCl (5 mL) and extracted with CH_2Cl_2 (3 x 5 mL). The combined organic fractions were dried over anhydrous $MgSO_4$, filtered and the solvent removed under reduced pressure. The resulting material was dissolved in a mixture of THF and H_2O (1:1, 1 mL) and an aqueous solution of NaOH (2.6 M, 0.50 mL) was added at room temperature. The reaction was stirred for 5 min before the addition of $Na_2S_2O_4$ (105 mg, 0.60 mmol). After a further 5 min, Me_2SO_4 (9.50 μL, 0.10 mmol) was added and the mixture stirred at room temperature for 1 h. The aqueous phase was extracted with CH_2Cl_2

(3 x 5 mL) and the combined organic fractions dried over anhydrous $MgSO_4$ and filtered. After removal of the solvent under reduced pressure the crude product was purified by flash column chromatography (SiO_2, 10-20% EtOAc in petroleum ether 40-60 °C) to yield the target compound (**16**) as a white solid (26 mg, 53%). m.p.: 85 °C; $[\alpha]_D$ –45.57 (*c* 0.10, $CHCl_3$).

IR ν_{max}/cm^{-1}:2963, 2874, 1721, 1651, 1157, 1063.

^{1}H NMR (400 MHz, $CDCl_3$) δ_H = 7.40 (s, 1H), 6.74 (s, 1H), 5.84 - 5.77 (m, 1H), 5.67 - 5.62 (m, 1H), 3.91 (s, 3H), 3.88 (s, 3H), 3.77 (s, 3H), 3.49 (ddd, *J* = 15.9, 4.8, 1.5 Hz, 1H), 3.13 - 3.06 (m, 1H), 3.04 (dd, *J* = 8.4, 6.8 Hz, 1H), 2.69 (d, *J* = 15.1 Hz, 1H), 2.57 (d, *J* = 1.4 Hz, 1H), 2.48 (ddd, *J* = 11.6, 8.6, 5.2 Hz, 1H), 2.28 (dd, *J* = 15.1, 1.8 Hz, 1H), 2.15 (td, *J* = 11.7, 6.7 Hz, 1H), 1.76 (dd, *J* = 11.8, 4.9 Hz, 1H), 1.55 (s, 9H), 1.16 - 1.06 (m, 1H), 1.04 - 0.94 (m, 1H) 0.63 (t, *J* = 7.5 Hz, 3H).

^{13}C NMR (100 MHz, $CDCl_3$) δ_C = 168.2, 151.3, 150.6, 148.4, 145.5, 134.3, 132.8, 129.6, 125.2, 111.9, 105.7, 101.9, 82.1, 68.1, 56.9, 56.2, 52.6, 51.7, 51.5, 51.2, 42.9, 41.6, 31.3, 28.3, 26.5, 7.7.

HRMS (EI^+): calculated for $C_{28}H_{36}N_2O_6$ $[M+H]^+$: m/z = 497.2652, m/z found = 497.2645.

1.2.8 Jerantinine A (1)[3]

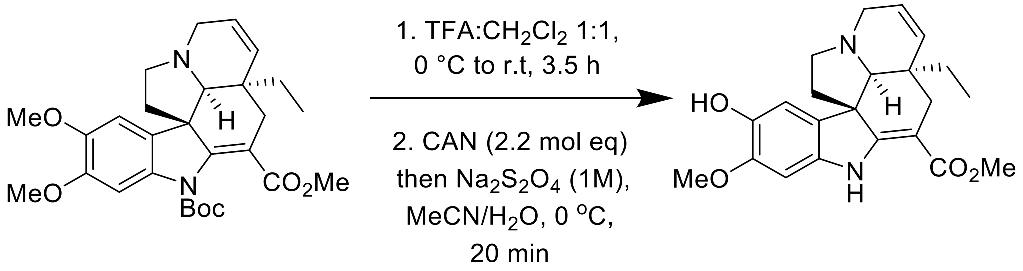

Scheme S-8. *Boc deprotection of N-boc-15,16-dimethoxy-tabersonine followed by demethylation with single electron oxidant (CAN) to synthesise jerantinine A.*

N-Boc-15,16-dimethoxy-tabersonine (50 mg, 0.10 mmol) was dissolved in a mixture of TFA and CH_2Cl_2 (1:1, 0.50 mL) at 0 °C and stirred. After 30 min the solution was warmed to room temperature and stirred for a further 3 h. The reaction mixture was poured into an aqueous solution of NaOH (2 M, 5 mL) at 0 °C and extracted with CH_2Cl_2 (3 x 5 mL). The combined organic fraction were then dried over anhydrous $MgSO_4$, filtered and the solvent removed under reduced pressure. To a stirred solution of crude 15,16-dimethoxy-tabersonine (40 mg, 0.10 mmol) in MeCN (3 mL) cooled to 0 °C was added a solution of ceric ammonium nitrate (120 mg, 0.22 mmol) in water (1 mL) at 0 °C. The reaction was stirred for 5 min and poured into a freshly prepared 1M solution of $Na_2S_2O_4$ in water (10 mL) at 0 °C. The reaction was stirred for a further 15 min before being diluted with CH_2Cl_2 (10 mL). The aqueous phase was extracted with CH_2Cl_2 (3 x 5 mL) and the organic fractions were combined. The organic fraction was dried over anhydrous $MgSO_4$, filtered and the solvent removed under reduced pressure to yield the crude product. The crude product was then purification by flash column chromatography (SiO_2, 10-20% EtOAc in petroleum ether 40-60 °C) yielded jerantinine A (**1**) as a white solid (26.0 mg, 72% over two steps). m.p.: 63.5 °C; $[\alpha]_D$ −23 (*c* 0.10, $CHCl_3$).

IR ν_{max}/cm^{-1}: 3548, 3379, 3035, 2965, 1669, 1605, 1110.

^{1}H NMR (500 MHz, $CDCl_3$) δ_H = 8.87 (br. s, 1H), 6.89 (s, 1H), 6.46 (s, 1H), 5.82 - 5.76 (m, 1H), 5.73 - 5.69 (m, 1H), 5.28 (br. s, 1H), 3.88 (s, 3H), 3.77 (s, 3H), 3.45 (ddd, J = 15.8, 4.6, 0.9 Hz, 1H), 3.20 - 3.12 (m, 1H), 3.06 - 3.00 (m, 1H), 2.68 (ddd, J = 11.2, 8.5, 4.7 Hz, 1H), 2.60 (s, 1H), 2.54 (dd, J = 15.0, 1.7 Hz, 1H), 2.42 (d, J = 15.0 Hz, 1H), 2.06 (td, J = 11.3, 6.4 Hz, 1H), 1.77 (dd, J = 11.6, 4.2 Hz, 1H), 1.04 - 0.95 (m, 1H), 0.91 – 0.82 (m, 1H), 0.64 (t, J = 7.5 Hz, 3H).

^{13}C NMR (125 MHz, $CDCl_3$) δ_C = 169.2, 168.0, 146.0, 140.1, 136.2, 133.2, 130.2, 125.1, 109.0, 94.6, 91.9, 70.3, 56.5, 55.4, 51.1, 51.0, 50.8, 44.5, 41.6, 28.5, 27.0, 7.6.

HRMS (EI^+): calculated for $C_{22}H_{27}N_2O_4$ $[M+H]^+$: m/z = 383.1971, m/z found = 383.1970.

Table S-1. Comparison of synthetic jerantinine A (**1**) NMR data with natural jerantinine A[3]

^{1}H NMR		^{13}C NMR	
**Kam *et al.***	**Synthetic jerantinine A (1)**	**Kam *et al.***	**Synthetic jerantinine A (1)**
0.64 (t, *J* = 7.3 Hz, 3H)	0.64 (t, *J* = 7.5, 3H)	7.4	7.6
0.86 (dq, *J* = 14.0, 7.3 Hz, 1H)	0.82 - 0.91 (m, 1H)	26.8	27.0
1.00 (dq, *J* = 14.0, 7.3 Hz, 1 H)	0.95 - 1.04 (m, 1H)	28.3	28.5
1.76 (dd, 11.5, 4.3 Hz, 1H)	1.77 (dd, *J* = 11.6, 4.2 Hz, 1H)	41.4	41.6
2.06 (td, *J* = 11.5, 7.0 Hz, 1H)	2.06 (td, *J* = 11.3, 6.4 Hz, 1H)	44.3	44.5
2.42 (d, *J* =15.0 Hz, 1H)	2.42 (d, *J* = 15.0 Hz, 1H)	50.6	50.8
2.53 (dd, *J* = 15.0, 1.5 Hz, 1H)	2.54 (dd, *J* = 15.0, 1.7 Hz, 1H)	50.8	51.0
2.60 (s, 1H)	2.60 (s, 1H)	50.9	51.1
2.68 (ddd, *J* = 11.5, 7.0, 4.3 Hz, 1H)	2.68 (ddd, *J* = 11.2, 8.5, 4.7 Hz, 1H)	55.2	55.4
3.02 (t, *J* = 7.0 Hz, 1H)	3.06 - 3.00 (m, 1H)	56.3	56.5
3.16 (br. d, *J* = 16.0 Hz, 1H)	3.20 - 3.12 (m, 1H)	70.1	70.3
3.45 (ddd, *J* = 16.0, 4.6, 1.3 Hz, 1H)	3.45 (ddd, *J* = 15.8, 4.6, 0.9 Hz, 1H)	91.6	91.9
3.76 (s, 3H)	3.77 (s, 3H)	94.4	94.6
3.87 (s, 3H)	3.88 (s, 3H)	108.8	109.0
5.36 (br. s, 1H)	5.28 (br. s, 1H)	124.8	125.1
5.70 (dt, *J* = 10.0, 1.3 Hz, 1H)	5.73 - 5.69 (m, 1H)	130.1	130.2
5.78 (ddd, *J* = 10.0, 4.6, 1.5 Hz, 1H)	5.82 - 5.76 (m, 1H)	133.0	133.2
6.45 (s, 1H)	6.46 (s, 1H)	135.9	136.2
6.89 (s, 1H)	6.89 (s, 1H)	139.9	140.1
8.87 (br. s, 1H)	8.87 (br. s, 1H)	145.8	146.0
		167.7	168.0
		169.2	169.2

1.2.9 Jerantinine E (3)[3]

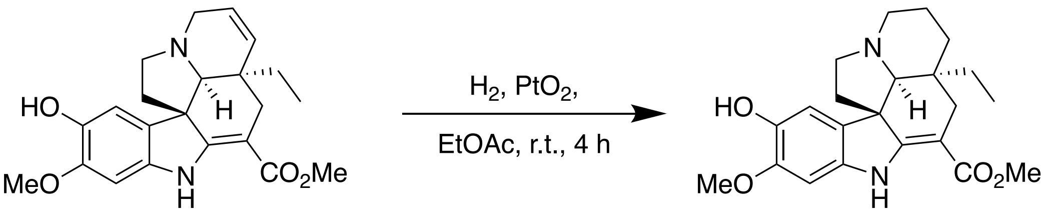

Scheme S-9. *Hydrogenation of jerantinine A to jerantinine E.*

To a solution of jerantinine A (6 mg, 0.016 mmol) in EtOAc (1 mL) was added PtO_2 (0.7 mg, 0.003 mmol) and the resulting mixture stirred under an atmosphere of H_2 for 4 h. The reaction was then filtered through Celite® before removal of the solvent under reduced pressure. Purification of the residue by flash column chromatography (SiO_2, 10% EtOAc in petroleum ether 40-60 °C) gave the desired product (**3**) (4.6 mg, 77%) as a colourless oil. $[\alpha]_D$ -351 (*c* 0.10, $CHCl_3$).

IR ν_{max}/cm^{-1}: 3535, 3365, 1668, 1602.

^{1}H NMR (500 MHz, $CDCl_3$) δ_H = 8.77 (br. s, 1H); 6.84 (s, 1H), 6.44 (s, 1H), 5.27 (br. s, 1H), 3.87 (s, 3H), 3.76 (s, 3H), 3.13 - 3.08 (m, 1H), 2.93 - 2.87 (m, 1H), 2.71 (d, *J* = 15.0 Hz, 1H), 2.57 - 2.49 (m, 1H), 2.42 - 2.35 (m, 2H), 2.26 (dd, *J* = 15.0, 1.7 Hz, 1H), 2.04 (td, *J* = 11.3, 6.4 Hz, 1H), 1.88 - 1.76 (m, 2H), 1.67 (dd, *J* = 11.4, 4.4 Hz, 1H), 1.57 - 1.50 (m, 1H), 1.27 - 1.24 (m, 1H) 1.03 - 0.93 (m, 1H), 0.67 - 0.56 (m, 4H).

^{13}C NMR (125 MHz, $CDCl_3$) δ_C = 169.5, 169.0, 145.8, 140.0, 136.3, 130.2, 108.6, 94.7, 92.5, 73.0, 56.5, 55.7, 51.8, 51.1, 50.9, 45.3, 38.4, 33.0, 29.4, 25.6, 22.4, 7.3.

HRMS (EI^+): calculated for $C_{22}H_{29}N_2O_4$ $[M+H]^+$: m/z = 385.2127, m/z found = 385.2130.

1.2.10 Jerantinine A acetate (7)[3]

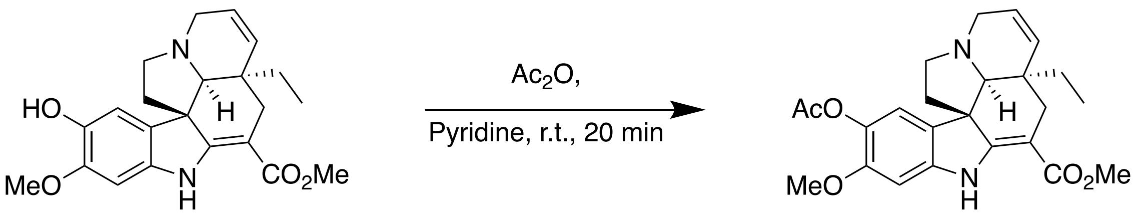

Scheme S-10. *Acetate protection of jerantinine A.*

Jerantinine A (**1**) (10 mg, 0.026 mmol) was dissolved in a mixture of Ac_2O and pyridine (1:1, 0.6 mL) and the resulting solution was stirred at room temperature for 20 min. The reaction mixture was then diluted with H_2O (3 mL) and basified to pH 9 with an aqueous 10% Na_2CO_3 solution. The mixture was then extracted with CH_2Cl_2 (3 x 5 mL) and the combined organic fraction dried over anhydrous $MgSO_4$, filtered and the solvent removed under reduced pressure. Purification of the residue by flash column chromatography (SiO_2, 10% EtOAc in petroleum ether 40-60 °C) gave the desired product as a colourless oil (**17**) (10.9 mg, 99%). $[\alpha]_D$ -222 (*c* 0.10, $CHCl_3$).

IR ν_{max}/cm^{-1}: 3257, 2955, 2918, 1637.

^{1}H NMR (400 MHz, $CDCl_3$) δ_H = 8.97 (s, 1H), 6.94 (s, 1H), 6.51 (s, 1H), 5.82 - 5.75 (m, 1H), 5.74 - 5.67 (m, 1H), 3.82 (s, 3H), 3.77 (s, 3H), 3.43 (dd, *J* = 15.8, 4.7 Hz 1H), 3.21 - 3.12 (m, 1H), 3.06 - 2.98 (m, 1H), 2.71 - 2.63 (m, 1H), 2.62 (s, 1H), 2.55 (dd, *J* = 15.1, 1.8 Hz, 1H), 2.40 (d, *J* = 15.1 Hz, 1H), 2.30 (s, 3H), 2.06 (td, *J* = 11.2, 6.6 Hz, 1H), 1.89 - 1.81 (m, 1H), 1.07 - 0.93 (m, 1H), 0.93 - 0.82 (m, 1H), 0.65 (t, *J* = 7.5z Hz, 3H).

^{13}C NMR (125 MHz, $CDCl_3$) δ_C = 169.6, 169.1, 167.1 151.1, 141.8, 133.6, 133.1, 129.7, 124.9, 116.5, 95.3, 92.9, 70.3, 56.4, 55.2, 51.2, 51.2, 50.5, 44.5, 41.2, 28.9, 28.3 27.3, 20.8, 7.6.

HRMS (EI^+): calculated for $C_{24}H_{29}N_2O_5$ $[M+H]^+$: m/z = 425.2076, m/z found = 425.2102.

2. Biological Data

2.1 Methods and Materials

2.1.1 Agent stocks

Jerantinine A (**1**), jerantinine A acetate (**7**) and colchicine were provided as solids and reconstituted with DMSO to yield concentrations of 10 mM. Stocks were stored as 10 μL aliquots at −80 °C protected from light.

2.1.2 Cell culture

Cells were passaged twice weekly upon reaching 70–80% confluency. Cells were sub-cultured in either RPMI 1640 medium containing sodium bicarbonate supplement (2.00 g/L), l-glutamine (0.30 g/L) and 10% heat-inactivated foetal bovine serum, or DMEM containing 10% heat-inactivated foetal bovine serum and 1% penicillin/streptomycin.

2.1.3 MTT assay

The 3-[4,5-dimethylthiazol-2-yl]-2,5-diphenyl tetrazolium bromide (MTT) assay, adapted from Mosmann[4], was used to assess the ability of test agents to inhibit cell growth and/or evoke cytotoxicity[5]. MTT assays were performed at the time of agent addition (T zero) and following 72 h exposure of cells to test agents, as previously described[6].

2.1.4 Clonogenic assay

The clonogenic cell survival test measures the ability of a single cell to survive brief exposure to test agents and maintain proliferative potential to form progeny colonies[7,8]. The assay was performed as previously described[6].

2.1.5 Cell cycle analysis

Cell cycle analysis was carried according to Nicoletti *et al.*[9] Cells were seeded in cell culture dishes at densities of 3–5 × 10^5 cells/dish in 10 mL medium. Following treatment, cells were harvested and pelleted by centrifugation then re-suspended in 0.5–1 mL fluorochrome solution (50 μg/mL propidium iodide (PI), 0.1 mg/ mL ribonuclease A, 0.1% v/v Triton X-100, and 0.1% w/v sodium citrate in dH_2O). Cells were stored overnight in

the dark at 4 °C. Cell cycle analyses were performed on a Beckman Coulter FC500 flow cytometer. EX-PO32 software was used to analyse data.

2.1.6 Tubulin polymerisation assay

The *in vitro* tubulin polymerisation assay[10-12] was conducted in 96-well plates according to the manufacturer's (Cytoskeleton, Inc) instructions. Incubations included 1 mM EGTA, 1 mM $MgCl_2$, tubulin buffer: 80 mM Na-PIPES (pH 6.9), test compound (jerantinine A, 1 μM; paclitaxel and nocodazole, 5 μM) and HTS-tubulin (4 mg/mL). Paclitaxel (microtubule-stabilising agent) and nocodazole (microtubule-destabilising agent) were included as positive and negative controls, respectively. Tubulin polymerisation was triggered by the addition of 1 mM GTP and carried out at 37 °C. The absorbance at 340 nm was measured over 1.15 h. Scattered light is directly proportional to the microtubule polymer concentration.

2.1.7 Confocal Microscopy

Confocal imaging was performed as previously described.[13] Procedures were performed at room temperature. Cells were fixed in formaldehyde (3.7% in PBS; 10–15 min) then permeabilised by PBT (PBS + 0.1% Triton X-100; 2–3 min). Blocking agent (PBT + 1% BSA; 1 h) was used to prevent binding non-specific protein binding. Cells were incubated with 1° Ab (monoclonal anti α-tubulin Ab, VWR International Ltd.; 2 h), washed with PBT before incubation in the dark with 2° Ab for 1 h. Cells were incubated with DNA binding dye (DRAQ5) for 5 min in the dark; a Zeiss LSM510 Meta confocal microscope was used to capture images.

2.1.8 3D culture

3D culture, imaging and quantification of MDA-MB-231 and MCF-7 cells were performed as previously described[14]. Briefly, cells were seeded on top of solidified Cultrex® (3433-005-01; Trevigen) and allowed to adhere for 60–90 min before 2% Cultrex in DMEM was overlaid. After three days of growth, jerantinine A (**1**), colchicine or vehicle (DMSO) were added in fresh medium to the 3D cultures. MDA-MB-231 cells were cultured in 5.36 μM

jerantinine A (**1**) or 10 μM colchicine. MCF-7 cells were cultured in 1.22 μM jerantinine A (**1**) or 14.5 μM colchicine. Imaging was completed at 0, 24 and 96 h post agent addition on the Olympus BX41 (40x magnification) or IX81 (100x magnification) microscopes. Quantification was completed using ImageJ, calculating the area for each colony. Multiple comparisons following one-way ANOVA of 96 h images post agent addition were calculated with GraphPad Prism v7.0.

2.2 Results.

2.2.1 Anti-cancer properties of the intermediates in the synthesis of jerantinine A, as determined by MTT assays

Table S-2. GI_{50} values for intermediates produced in the synthesis of jerantinine A (**1**) and jerantinine A acetate (**7**). Cells were treated with concentrations of test agents (5 nM – 50 μM; n = 4 per trial). At the time of agent addition, and following 72 h exposure MTT assays were performed. Data are mean ± SEM ≥3 independent experiments.

Compound	GI_{50} values (μM)	
	MCF-7	HCT116
	>50	-
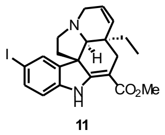 **11**	>50	-
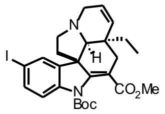 **12**	6.52±0.11	-
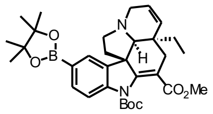 **13**	>50	-

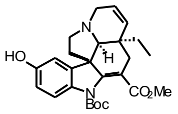 **14**	>50	-
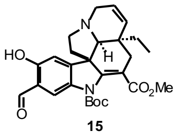 **15**	3.35±0.07	-
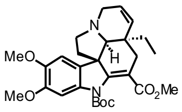 **16**	>50	-
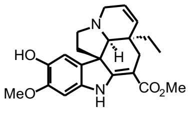 **1**	0.809±0.074	0.819±0.072
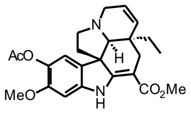 **7**	0.38±0.01	0.350±0.025
**Colchicine**		0.031±0.001

2.2.2 Cell cycle analysis

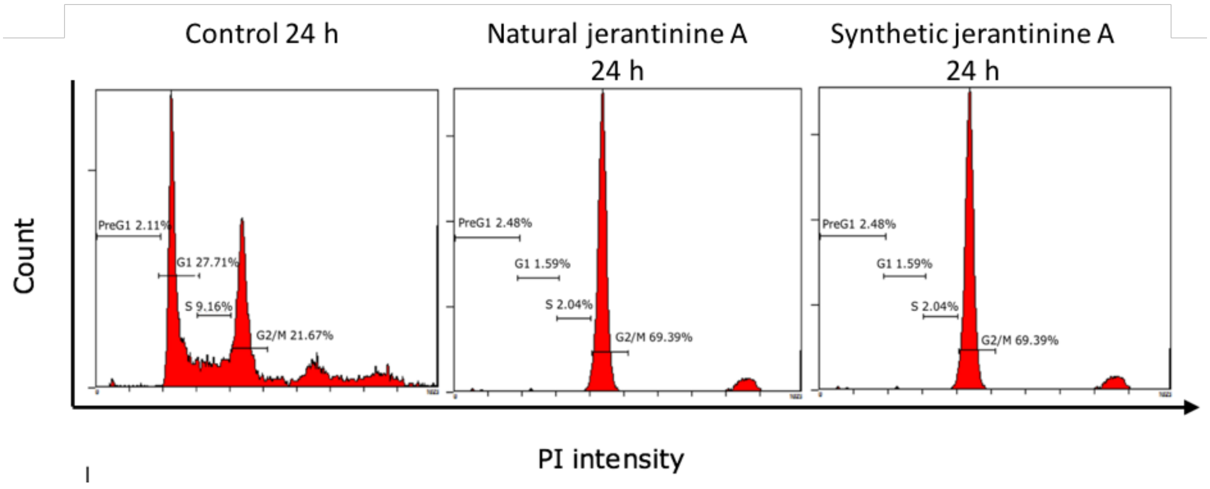

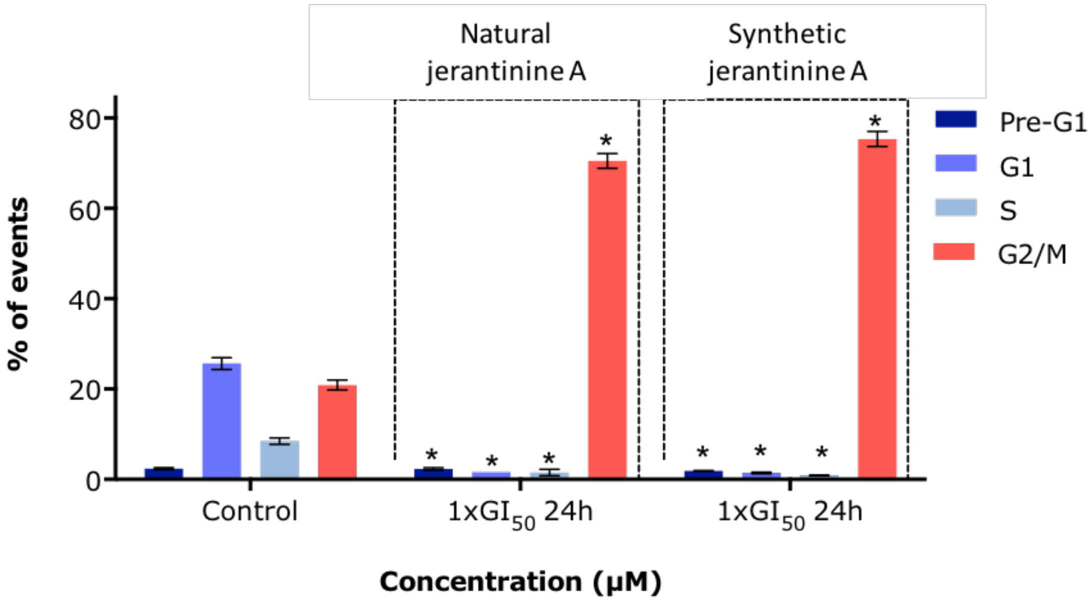

Figure S-1. Representative cell cycle histograms and collated graphical illustration of HCT-116 cell cycle distribution of singlet events) following exposure of cells to jerantinine A (**1**) or vehicle control (24 h). Analyses were performed on 3 separate occasions, in duplicate; 20,000 events were analysed per sample.

2.2.3 Confocal Microscopy

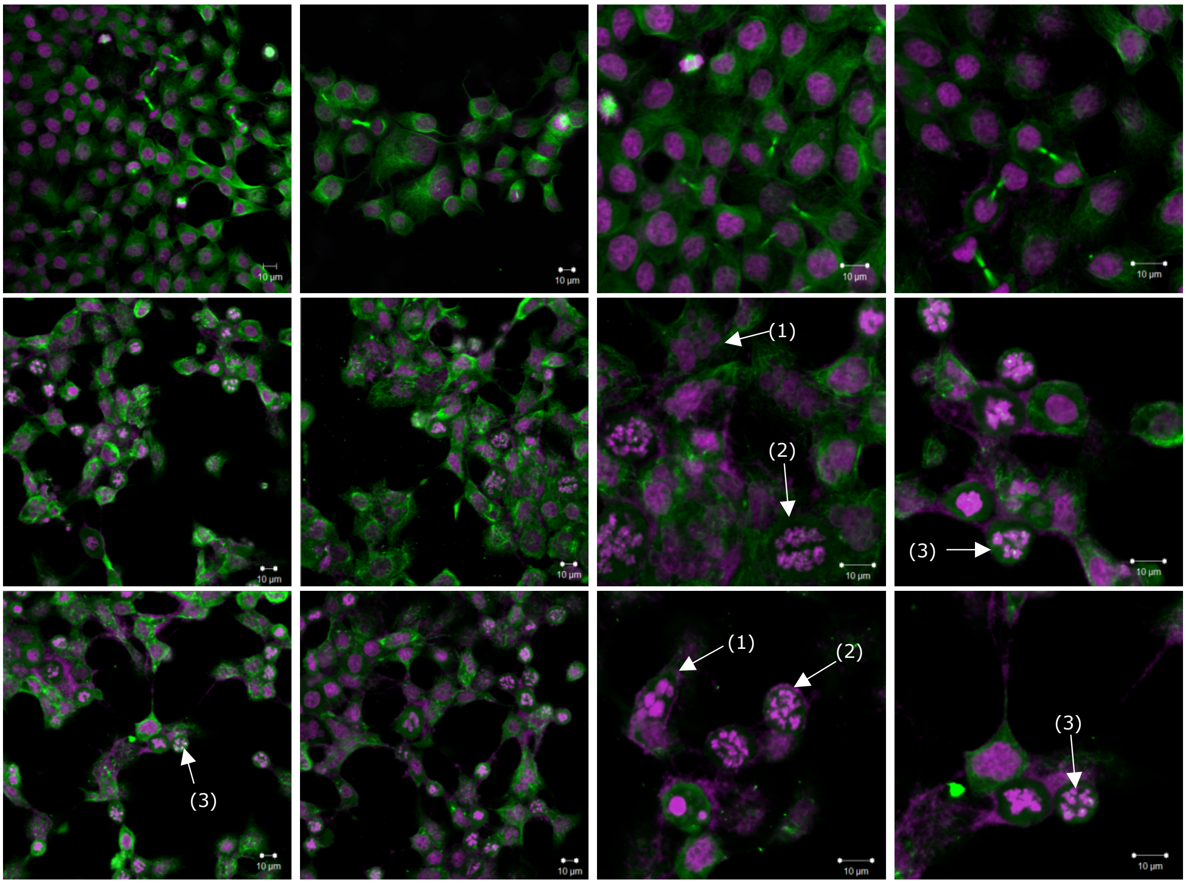

Figure S-2. Effects of synthetic jerantinine A (**1**) and synthetic jerantinine A acetate (**7**) (24 h exposure) on MCF-7 cell morphology. 1st row: controls with vehicle only; 2nd row: jerantinine A (1x GI_{50} = 0.809 µM); 3rd row: jerantinine A acetate (1x GI_{50} = 0.378 µM). Jerantinine A and jerantinine A acetate caused multinucleation (1), nuclear fragmentation (2) and multipolar spindle formation (3).

2.3 Analysis

2.3.1 MTT assay

Jerantinine A (**1**) and jerantinine A acetate (**7**) had GI_{50} values of ~ 0.80 and 0.30 μM in MCF-7 and HCT116 respectively. The GI_{50} values for most intermediates were over >50 μM. However, **12** and **15** exhibited some activity against MCF-7 cells with GI_{50} values of 6.52 and 3.35 μM, respectively.

2.3.2 Cell cycle analysis

MTT and clonogenic assays suggest that jerantinine A (**1**) compromises cancer cell growth and viability. Guided by these observations, we investigated the effect of jerantinine A (**1**) on cell cycle perturbation by flow cytometry. HCT 116 cells were treated with natural and synthetic jerantinine A (**1**) at 1x GI_{50} for 24 h. Stark G2/M cell cycle arrest was observed following 24 h exposure to natural and synthetic jerantinine A.

2.3.3 Jerantinine A and jerantinine A acetate inhibit tubulin polymerisation

Profound G2/M cell cycle arrest led us to investigate the effect of synthetic jerantinine A (**1**) and jerantinine A acetate (**7**) on tubulin polymerisation and compare effects to natural jerantinine A, tubulin stabilising and destabilising agents paclitaxel and nocodazole respectively (Figure 3B). Paclitaxel (5 μM) induced rapid polymerisation of tubulin. In contrast, 5 μM nocodazole arrested tubulin polymerisation. The effect of synthetic jerantinine A (**1**) and jerantinine A acetate (**7**) on tubulin polymerisation was unequivocal and indistinguishable from natural jerantinine A and nocodazole: synthetic jerantinine A (**1**) and jerantinine A acetate (**7**) (1 μM) comprehensively inhibited tubulin polymerisation.

2.3.4 Confocal Microscopy

After 24 h of treatment, extensive mitotic disruption was observed. Multinucleation, nuclear fragmentation and multipolar spindles were clearly evident in treated cells exclusively; the morphology of non-treated cells demonstrated normal cell division.

2.3.5 Discussion

Jerantinine A (**1**) and jerantinine A acetate (**7**) are *aspidosperma* indole alkaloids, and have shown profound growth inhibitory and cytotoxic activity against human-derived MCF-7 and HCT-116 cancer cell lines. Jerantinine A acetate (**7**) had slightly higher potency, and would represent a better drug candidate due to its increased stability. Additionally, the presence of an acetate group reduces overall polarity and could help intracellular access across hydrophobic cell membranes.

Confocal microscopy, following treatment of cells with synthetic jerantinine A (**1**) and jerantinine A acetate (**7**) was undertaken. The DNA and tubulin were stained to show any changes in cytoskeletal architecture and cell morphology compared to untreated cells. Treatment of tumour cells with jerantinine A (**1**) and jerantinine A acetate (**7**) led to cytoskeletal malformations characteristic of failed tubulin polymerisation. These include multipolar spindles and irregular chromosome segregation; in addition apoptotic signs such as DNA fragmentation were observed. Confocal microscopy images reveal many examples of multipolar spindles and misaligned chromosomes (Figure S-2), multinucleation (aneuploidy) and nuclear fragmentation. Results of cell cycle analyses and tubulin polymerisation assays concur: synthetic jerantinine A (**1**) and jerantinine A acetate (**7**) caused profound G2/M cell cycle arrest, a consequence of tubulin polymerisation microtubule disarray and formation of multipolar spindles.

3. X-ray crystallography data

3.1 Crystallisation, data collection and structure determination

Crystals of T_2R-TTL were generated as described previously[15,16]. Suitable T_2R-TTL crystals were incubated 30 min, 1 h or 3 h in a reservoir solution containing 10% PEG 4K, 12% glycerol and 5 mM of jerantinine B acetate, and were flash cooled in liquid nitrogen following a brief transfer into a cryo solution containing 18% glycerol.

X-ray diffraction data were collected at beamline X06SA at the Swiss Light Source (Paul Scherrer Institut, Villigen PSI, Switzerland). Images were indexed and processed using XDS[17]. Structure solution using the difference Fourier method and refinement were performed using the PHENIX package[18]. Model building was carried out iteratively using the Coot software[19]. PyMOL was used for figure preparation (The PyMOL Molecular Graphics System, Version 1.8.6.0. Schrödinger, LLC). Data collection and refinement statistics for the T_2R-TTL-JBA are given in Table S-3.

Table S-3. Related to Figure S-3. X ray crystallography data collection and refinement statistics.

**Data collection**[a]	**T_2R-TTL-Jerantinine B acetate**
**Space group**	$P2_12_12_1$
**Cell dimensions**	
a, b, c (Å)	104.6, 157.2, 180.1
Resolution (Å)[b]	49.4 – 2.4 (2.46 – 2.40)
R_{meas} (%)	15.9 (410.1)
R_{pim} (%)	
CC_{half}[c]	99.9 (20.4)
$\langle I \rangle / \langle \sigma I \rangle$	12.5 (0.7)
Completeness (%)	99.7 (99.6)
Redundancy	12.7 (12.8)
**Refinement**	
Resolution (Å)	48.0 – 2.4
No. unique reflections	117393 (5877 in test set)
R_{work} / R_{free} (%)	19.9 / 25.0
**Average B-factors (Å²)**	
complex	84.7
solvent	66.9
Compound (chain B)	71.2
Compound (chain D)	-
Wilson B-factor	63.9
**Root mean square deviation from ideality**	
Bond length (Å)	0.003
Bond angles (°)	0.554
**Ramachandran statistics**[d]	
Favoured regions (%)	96.6
Allowed regions (%)	3.35
Outliers (%)	0.05

[a] Highest shell statistics are in parentheses. [b] Resolution cut-offs were chosen based on CC_{half} and $\langle I \rangle / \langle \sigma I \rangle$ as described by Karplus & Diederichs[20]. [c] As defined by Karplus & Diederichs[20]. [d] As defined by MolProbity[21].

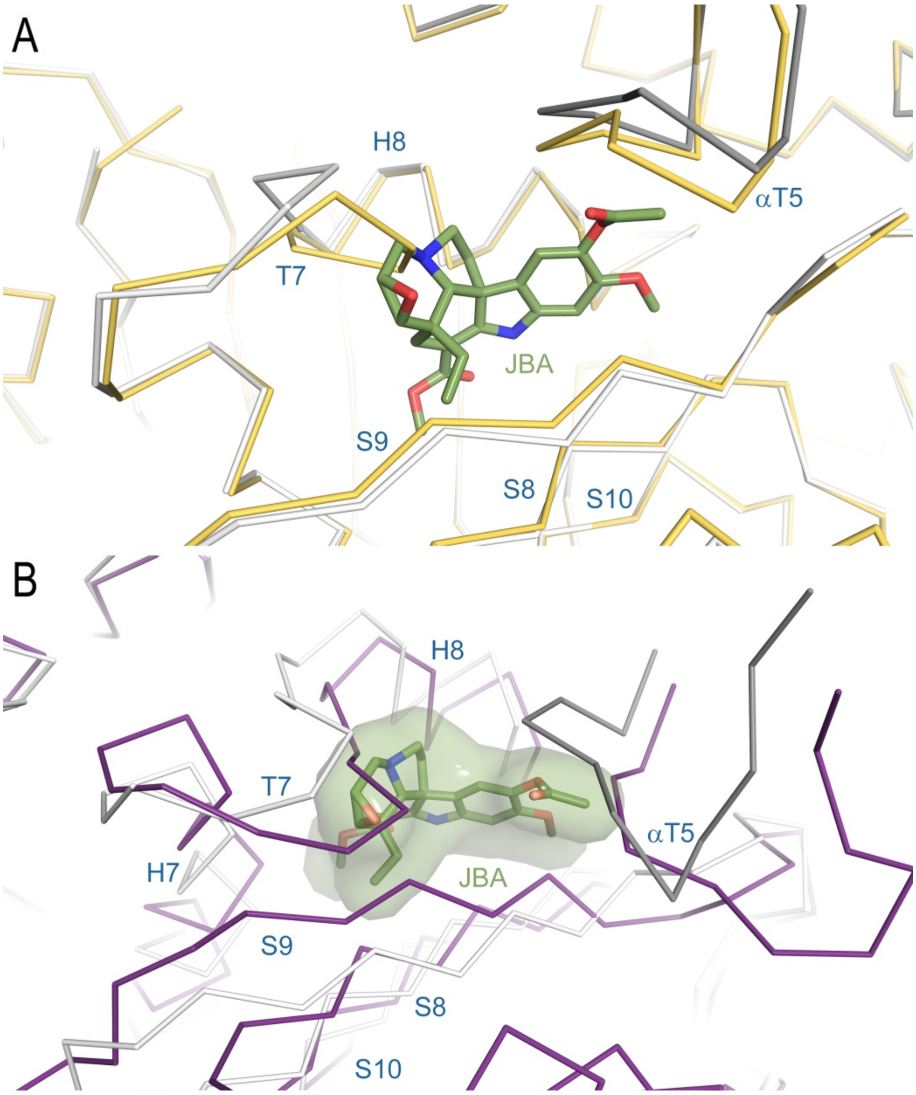

Figure S-3. Structural changes upon interaction of jerantinine B acetate (**9**) with tubulin and jerantinine B acetate (**9**) binding in the context of a microtubule.

(A) Ribbon representation of the superimposed apo (yellow, PDB ID 4I55) and tubulin-bound jerantinine B acetate (light and dark grey) structures highlighting the conformational changes that occur upon ligand binding at both the αT5- and βT7-loops. The secondary structural elements are labelled in blue. The jerantinine B acetate (**9**) is in green sticks representation. The structures were superimposed onto their β1-tubulin chains (chain B of T_2R-TTL, rmsd of 0.24 Å over 351 Cα-atoms).

(B) Jerantinine B acetate (**9**) binding in the context of a microtubule. Ribbon representation of the superimposed N- and C-terminal domains of β-tubulin in the microtubule (violet-purple, PDB ID 5SYF, rmsd of 0.61 Å over 226 Cα-atoms) and the corresponding domain in tubulin-jerantinine B acetate (light and dark grey) structures highlighting the structural rearrangements of the αT5-loop helices βH7 and βH8, the βT7-loop, and both the β-strands βS8 and βS9, which occur upon the "curved-to-straight" tubulin conformational transition accompanying microtubule assembly[22]. These conformational changes result in a compaction of the colchicine site, a process that is inhibited in the presence of a bound ligand[23].

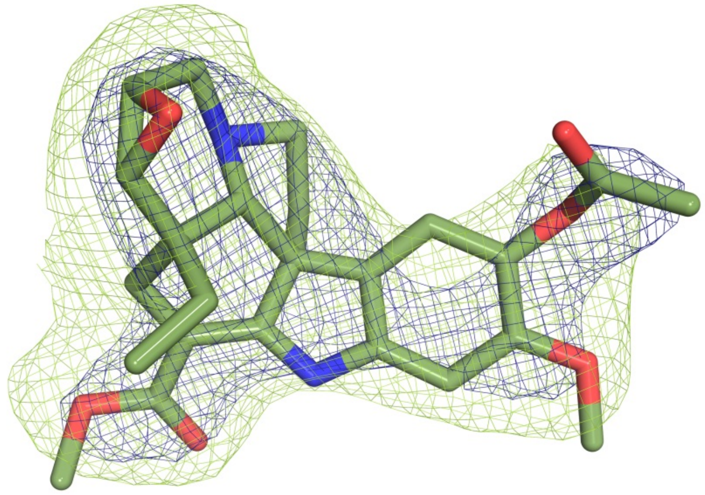

Figure S-4. Simulated annealing omit map. The SigmaA-weighted 2mFo-DFc (dark blue mesh) and mFo-DFc (light green mesh) electron density omit maps are contoured at +1.0σ and +3.0σ, respectively. The maps were calculated excluding the atoms corresponding to jerantinine B acetate (**9**) only.

4. Modelling of Binding Studies

4.1 Methods

The X-ray crystal structures of the colchicine-tubulin complex (PDB ID 4o2b)[24], the vinblastine-tubulin complex (PDB ID 5j2t)[25], the structure of the taxol-tubulin complex determined by electron crystallography (PDB ID 1jff)[26], and the structure of the (-)-jerantinine B acetate-tubulin complex determined here, were used in *in silico* docking studies. Comparative modelling was performed on the tubulin structures to introduce the human tubulin amino acid sequence using the MODELLER software[27]. Ligand structures were minimised at the B3LYP/6-31G(d) level using the GAUSSIAN-03 software[28]. Docking was performed using the Vina software,[29] implemented in YASARA (www.yasara.com). The highest-scoring docking solutions from the Vina analysis were subjected to local geometry optimisation; the binding energies from these calculations are reported in Table 2.

4.2 Results

The structure of both α– and β-tubulin comprises six alpha helices (H1-H6), six beta strands (S1-S7), and six loops (T1-T6) connecting alternating helices and strands[26]. Taxol binds only β-tubulin, interacting with residues in helix H1, the H6-H7 loop, the H7 helix, the S7-H9 loop, and the M-loop - this is often referred to as the taxane site; binding to this site results in microtubule stabilisation. On α-tubulin, vinblastine interacts with residues in H10, S9 and the T7-loop, and on β-tubulin with residues in the T5-loop and H6-H7 loop. Ligands binding to the vinblastine site also destabilise microtubules. Jerantinine B acetate binds the colchicine binding pocket, also at the interface between α– and β-tubulin subunits; this pocket is formed by residues in the T5-loop on α-tubulin, and H7, H8, S8, S9 and the T7-loop on β-tubulin. Thus, the three sites, taxane-, colchicine- and vinblastine-, are distinct and non-overlapping[30].

Table S4. Predicted binding energies (kJ mol^{-1})

Binding site:	Colchicine	Vinblastine	Taxol	RMSD[a]
**Ligand:**				
Colchicine	44.2	32.9	29.6	0.74
(-)-Jerantinine A (**1**)	45.6	32.8	30.7	
(-)-Jerantinine E (**3**)	45.2	33.2	31.5	
(-)-Tabersonine (**4**)	43.7	32.5	30.5	
16-methoxytabersonine (**5**)	45.0	34.5	30.7	
(-)-Melodinine P (**6**)	44.4	33.6	31.4	
(-)-Jerantinine A acetate (**7**)	48.3	31.2	30.6	
(-)-Jerantinine B (**8**)	46.8	33.2	31.3	
(-)-Jerantinine B acetate (**9**)	49.3	34.3	31.0	0.68

[a] Root mean square deviation of ligand non-hydrogen atoms between experimental structure and Vinca docked geometry bound to their cognate binding site (Å).

5. References

1. Zhao, S. & Andrade, R. B. Domino Michael/Mannich/ N-alkylation route to the tetrahydrocarbazole framework of Aspidosperma alkaloids: Concise total syntheses of (-)-aspidospermidine, (-)-tabersonine, and (-)-vincadifformine. *J. Am. Chem. Soc.* **135,** 13334–13337 (2013).

2. Chen, F., Lei, M. & Hu, L. Synthesis of C-10 tabersonine analogues by palladium-catalyzed cross-coupling reactions. *Synth.* **46,** 3199–3206 (2014).

3. Lim, K. H., Hiraku, O., Komiyama, K. & Kam, T. S. Jerantinines A-G, cytotoxic Aspidosperma alkaloids from Tabernaemontana corymbosa. *J. Nat. Prod.* **71,** 1591–1594 (2008).

4. Mosmann, T. Rapid colorimetric assay for cellular growth and survival: Application to proliferation and cytotoxicity assays. *J. Immunol. Methods* **65,** 55–63 (1983).

5. Morgan, D. M. Tetrazolium (MTT) assay for cellular viability and activity. *Methods Mol. Biol.* **79,** 179–183 (1998).

6. Raja, V. J., Lim, K. H., Leong, C. O., Kam, T. S. & Bradshaw, T. D. Novel antitumour indole alkaloid, Jerantinine A, evokes potent G2/M cell cycle arrest targeting microtubules. *Invest. New Drugs* **32,** 838–850 (2014).

7. Munshi, A., Hobbs, M. & Meyn, R. E. In Vitro Measures of Chemosensitivity. *Methods Mol. Med.* **110,** 21–28 (2005).

8. Plumb, J. A. Cell sensitivity assays : clonogenic assay. *Methods Mol. Med*. **88,** 159–164 (1999).

9. Nicoletti, I., Migliorati, G., Pagliacci, M. C., Grignani, F. & Riccardi, C. A Rapid and Simple Method for Measuring Thymocyte Apoptosis By Propidium Iodide Staining and Flow-Cytometry. *J. Immunol. Methods* **139,** 271–279 (1991).

10. Shelanski, M. L., Gaskin, F. & Cantor, C. R. Microtubule assembly in the absence of added nucleotides. *Proc. Natl. Acad. Sci. U. S. A.* **70,** 765–8 (1973).

11. Lee, J. C. & Timasheff, S. N. In vitro reconstitution of calf brain microtubules: effects of solution variables. *Biochemistry* **16,** 1754–1764 (1977).

12. Lam, F. et al. ZJU-6, a novel derivative of Erianin, shows potent anti-tubulin polymerisation and anti-angiogenic activities. *Invest. New Drugs* **30,** 1899–1907 (2012).

13. Collins, H. M. *et al.* Differential effects of garcinol and curcumin on histone and p53 modifications in tumour cells. *BMC Cancer* **13,** 37 (2013).

14. Duivenvoorden, H. M. *et al*. Myoepithelial cell-specific expression of stefin A as a suppressor of early breast cancer invasion. *J Pathol.* **243,** 496-509 (2017).

15. Prota, A. E. *et al.* Structural basis of tubulin tyrosination by tubulin tyrosine ligase. *J. Cell Biol.* **200,** 259–270 (2013).

16. Prota, A. E. *et al.* Molecular Mechanism of Action of Microtubule-Stabilizing Anticancer agents. *Science* **339,** 587 (2013).

17. Kabsch, W. XDS. *Acta Crystallogr. Sect. D Biol. Crystallogr.* **66,** 125–132 (2010).

18. Adams, P. D. *et al.* PHENIX: A comprehensive Python-based system for macromolecular structure solution. *Acta Crystallogr. Sect. D Biol. Crystallogr.* **66,** 213–221 (2010).

19. Emsley, P. & Cowtan, K. Coot: Model-building tools for molecular graphics. *Acta Crystallogr. Sect. D Biol. Crystallogr.* **60,** 2126–2132 (2004).

20. Karplus, P. A. & Diederichs, K. Linking Crystallographic Model and Data Quality. *Science* **336,** 1030–1033 (2012).

21. Davis, I. W., Murray, L. W., Richardson, J. S. & Richardson, D. C. MolProbity: Structure validation and all-atom contact analysis for nucleic acids and their complexes. *Nucleic Acids Res.* **32,** 615–619 (2004).

22. Ravelli, R. B. G. *et al.* Insight into tubulin regulation from a complex with colchicine and a stathmin-like domain. *Nature* **428,** 198 (2004).

23. Dorléans, A. *et al.* Variations in the colchicine-binding domain provide insight into the structural switch of tubulin. *Proc. Natl. Acad. Sci. U. S. A.* **106,** 13775–9 (2009).

24. Prota, A. E. et al. The novel microtubule-destabilizing drug BAL27862 binds to the colchicine site of tubulin with distinct effects on microtubule organization. *J. Mol. Biol.* **426,** 1848–1860 (2014).

25. Waight, A. B. *et al.* Structural basis of microtubule destabilization by potent auristatin anti-mitotics. *PLoS One* **11,** 1–14 (2016).

26. Löwe, J., Li, H., Downing, K. & Nogales, E. Refined structure of αβ-tubulin at 3.5 Å resolution. *J. Mol. Biol.* **313,** 1045–1057 (2001).

27. Webb, B. & Sali, A. Comparative protein structure modeling using MODELLER. *Curr. Protoc. Bioinforma.* 5.6.1-5.6.32 (2014).

28. Frisch, M. J. et al. Guassian 09, Revision E.01. Guassian, Inc., Wallingford CT (2013).

29. Trott, O. & Olson, A. J. Valence Bond Theory for Chemical Dynamics. *J. Comput. Chem.* **2831,** 455–461 (2009).

30. Stanton, R. A., Gernert, K. M., Nettles, J. H. & Aneja, R. Drugs that target dynamic microtubules: A new molecular perspective. *Med. Res. Rev.* **31,** 443–481 (2011).

6. Spectroscopic Data

Tabersonine ¹H NMR (4)

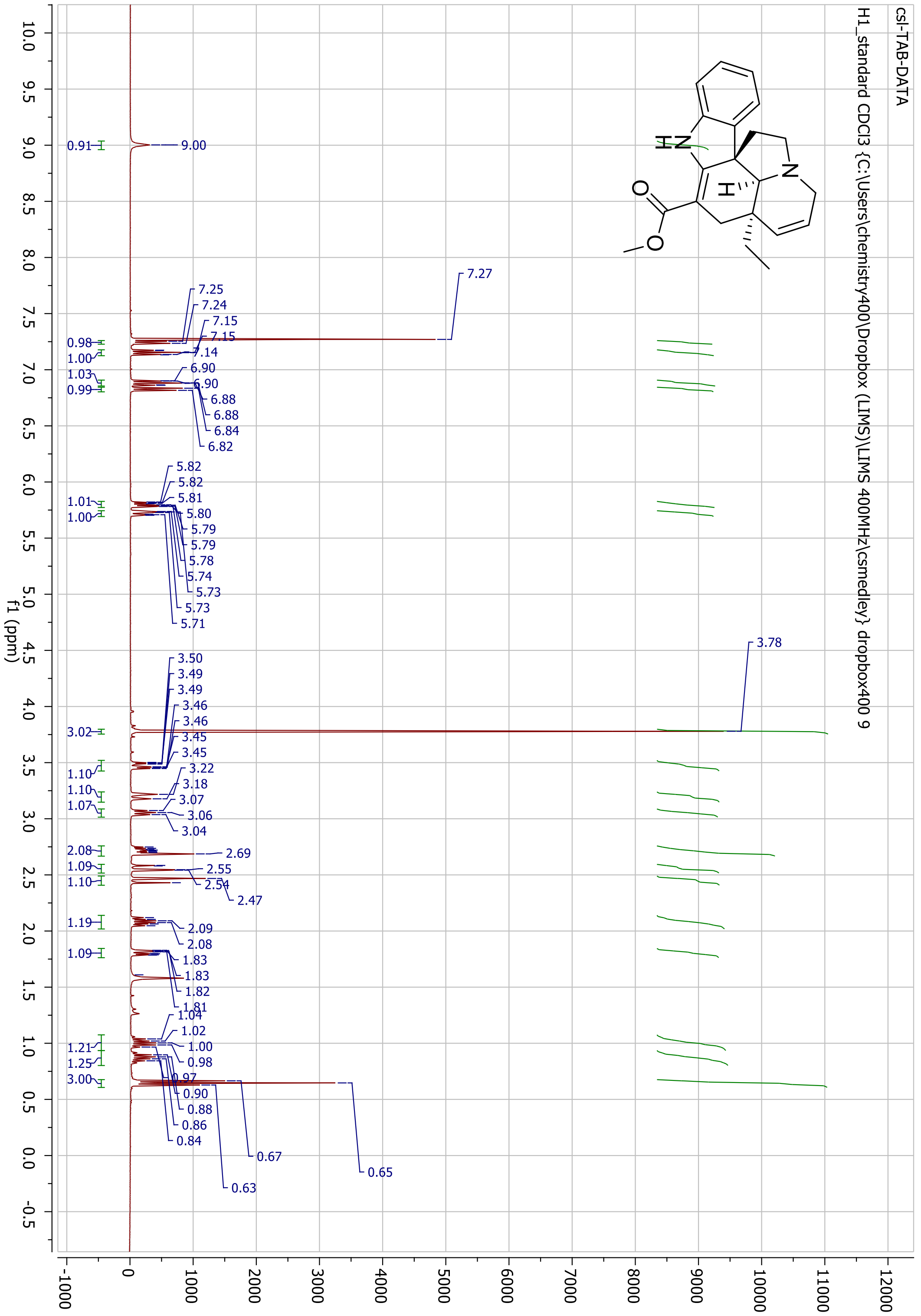

Tabersonine ^{13}C NMR (4)

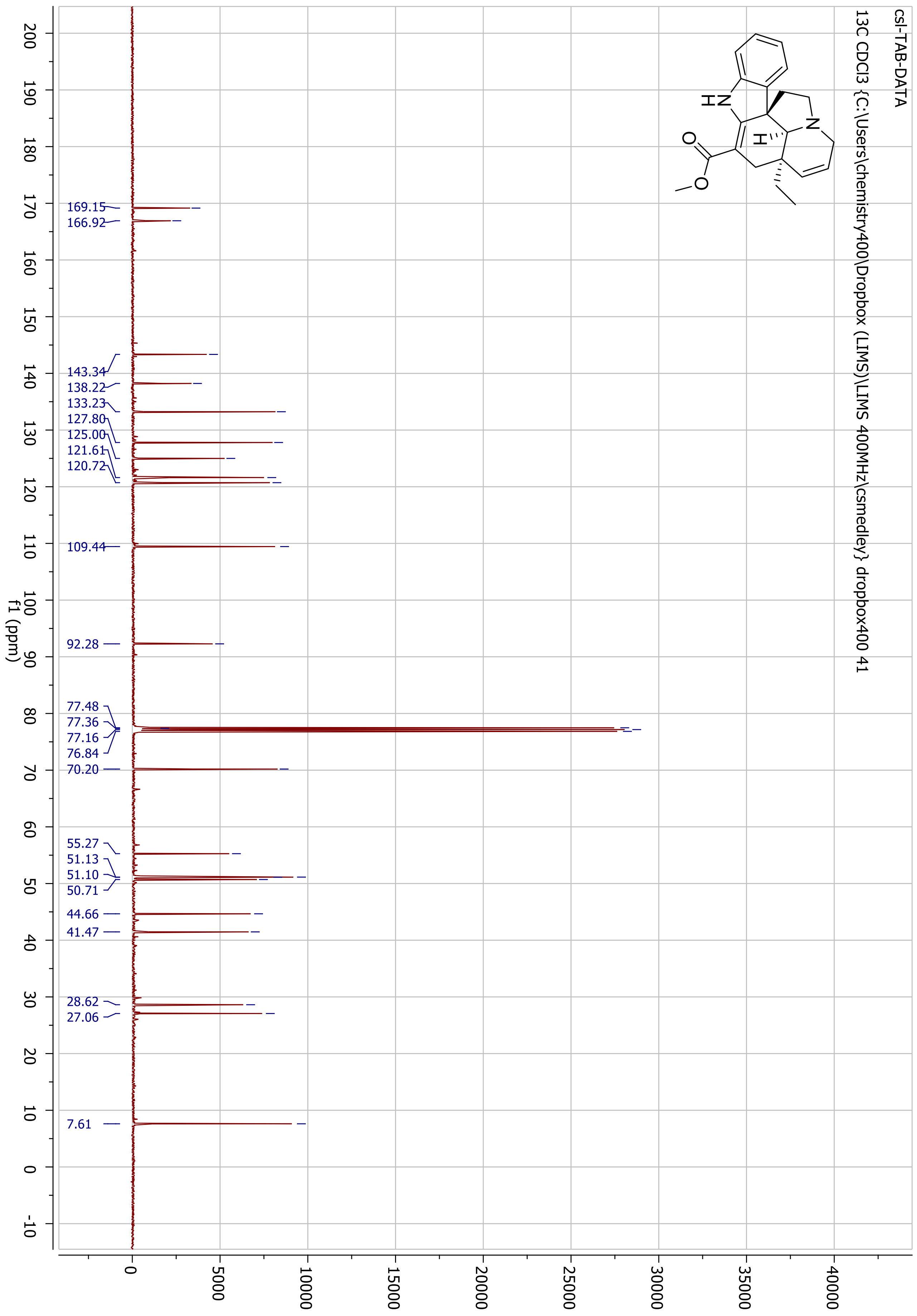

15-Iodo-Tabersonine ^{1}H NMR (11)

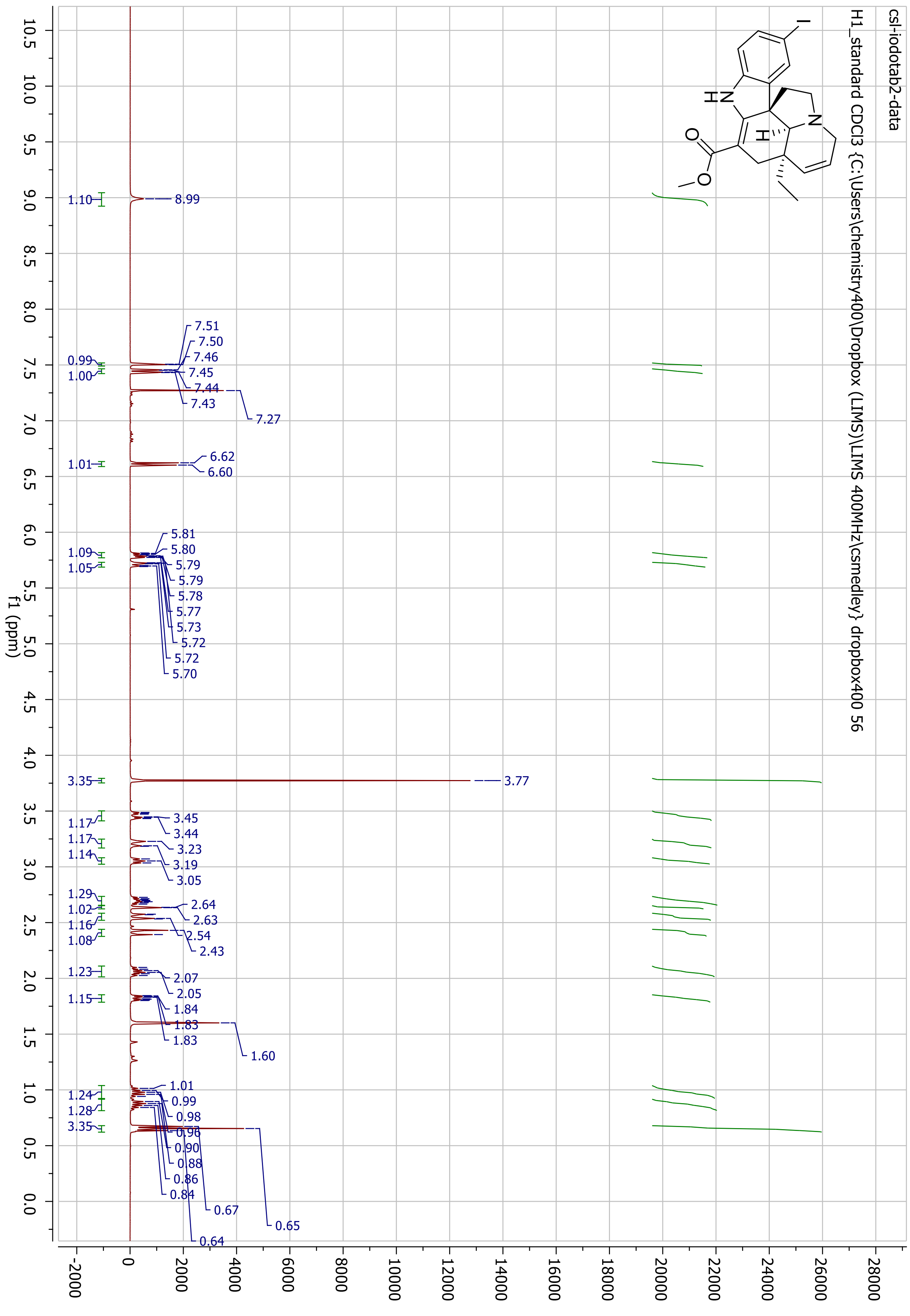

15-Iodo-Tabersonine ^{13}C NMR (11)

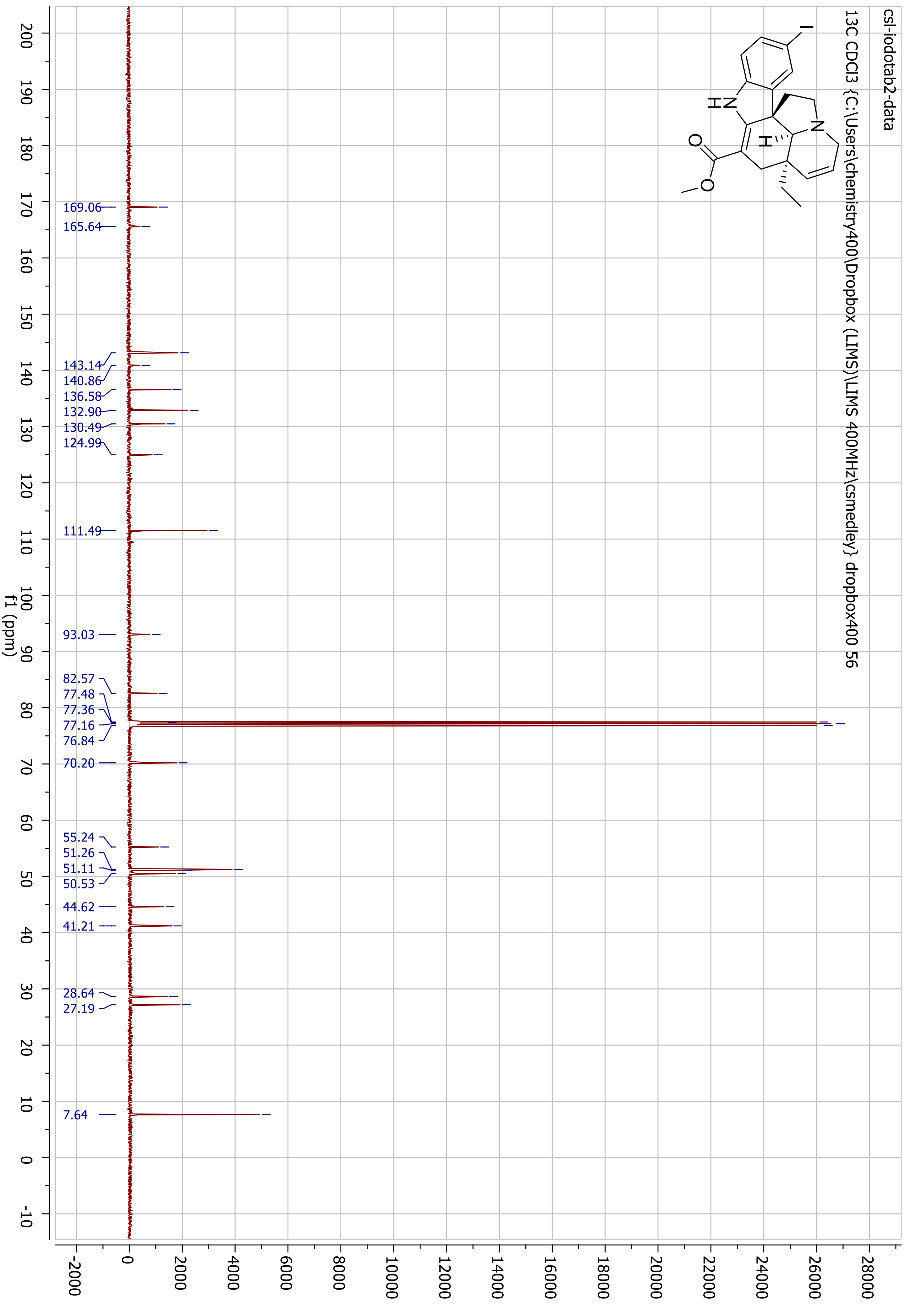

_N_-Boc-15-iodo-tabersonine ^{1}H NMR (12)

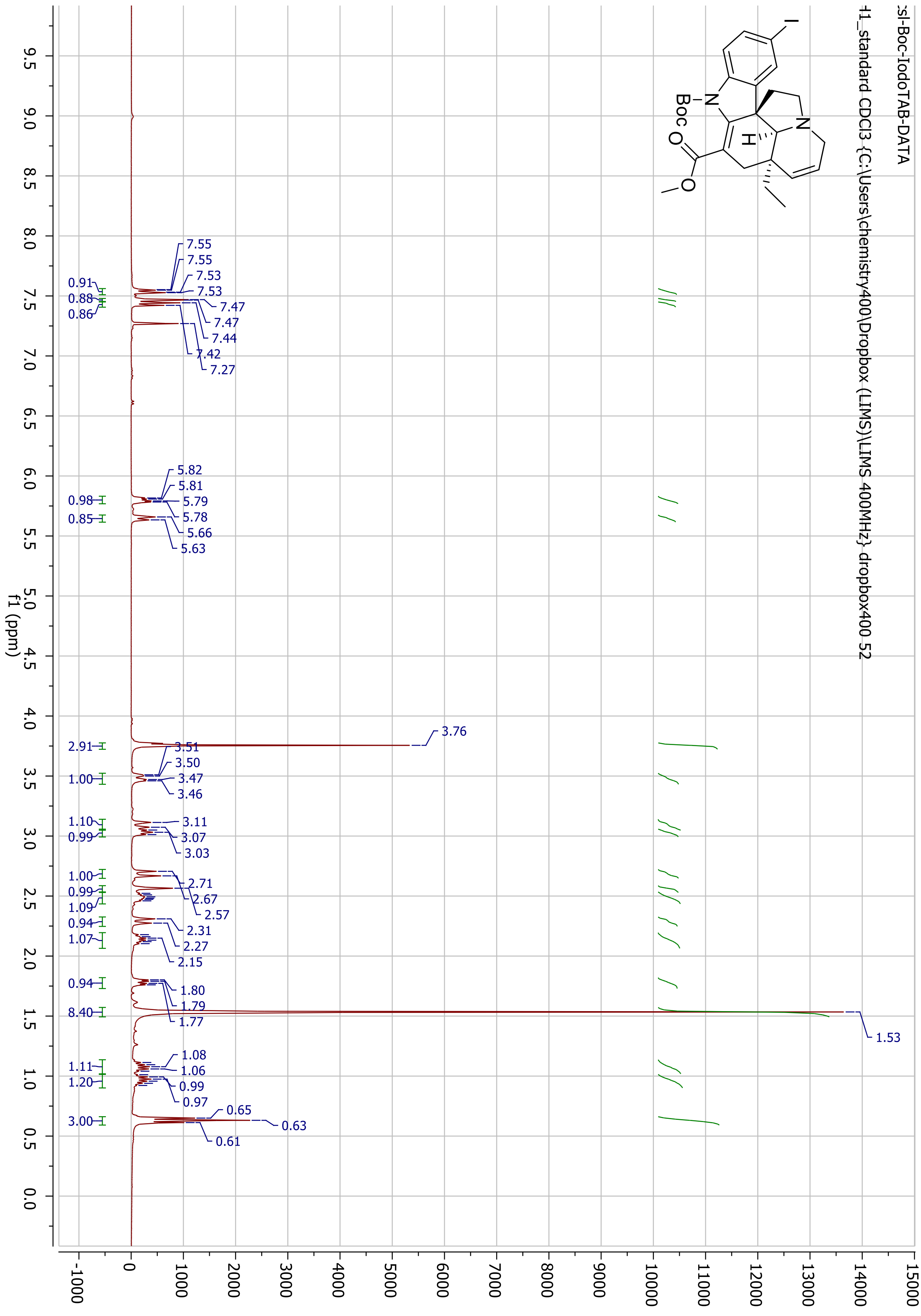

***N*-Boc-15-iodo-tabersonine ^{13}C NMR (12)**

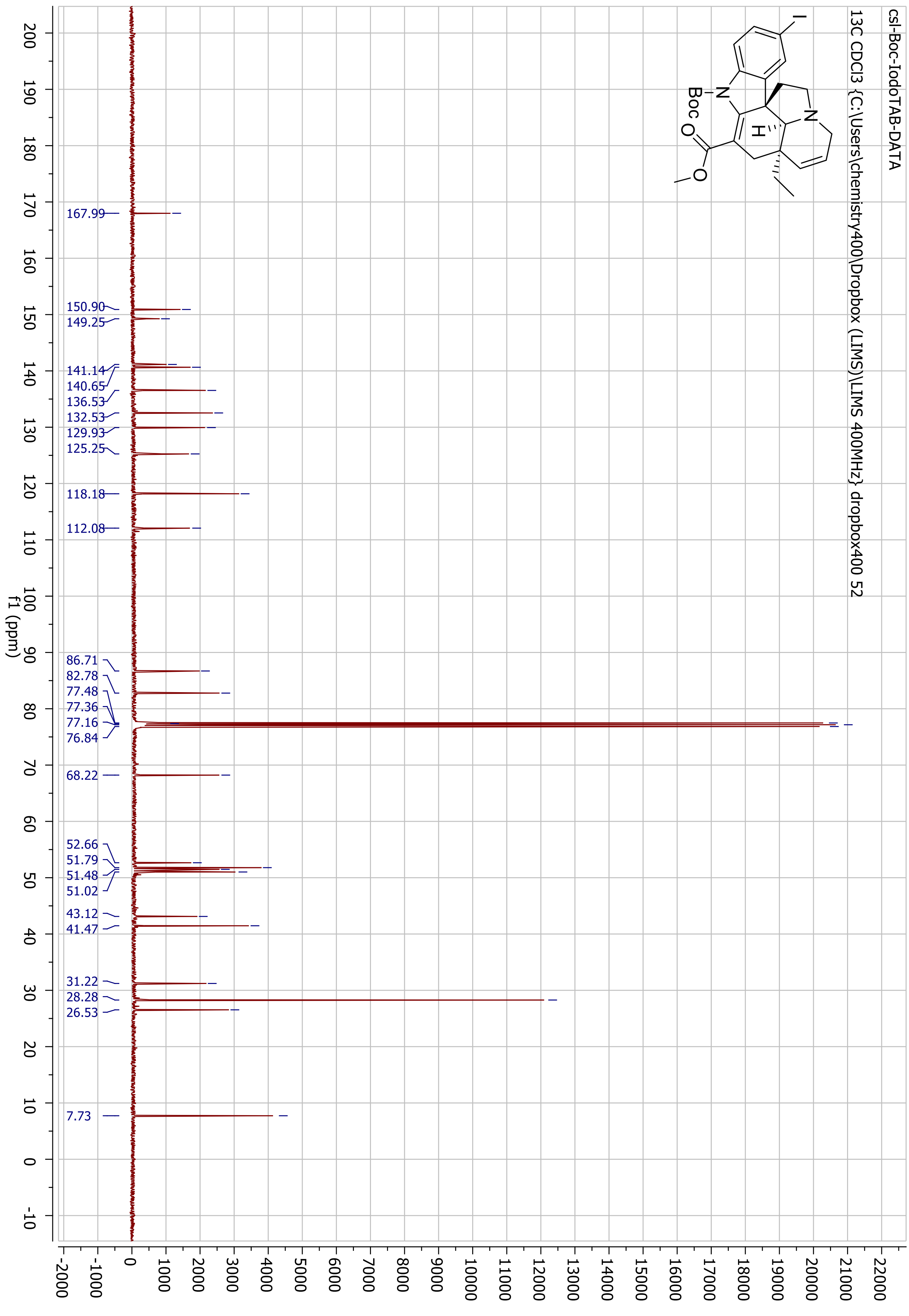

***N*-Boc-tabersonine-15-boronic acid pinacol ester ^{1}H NMR (13)**

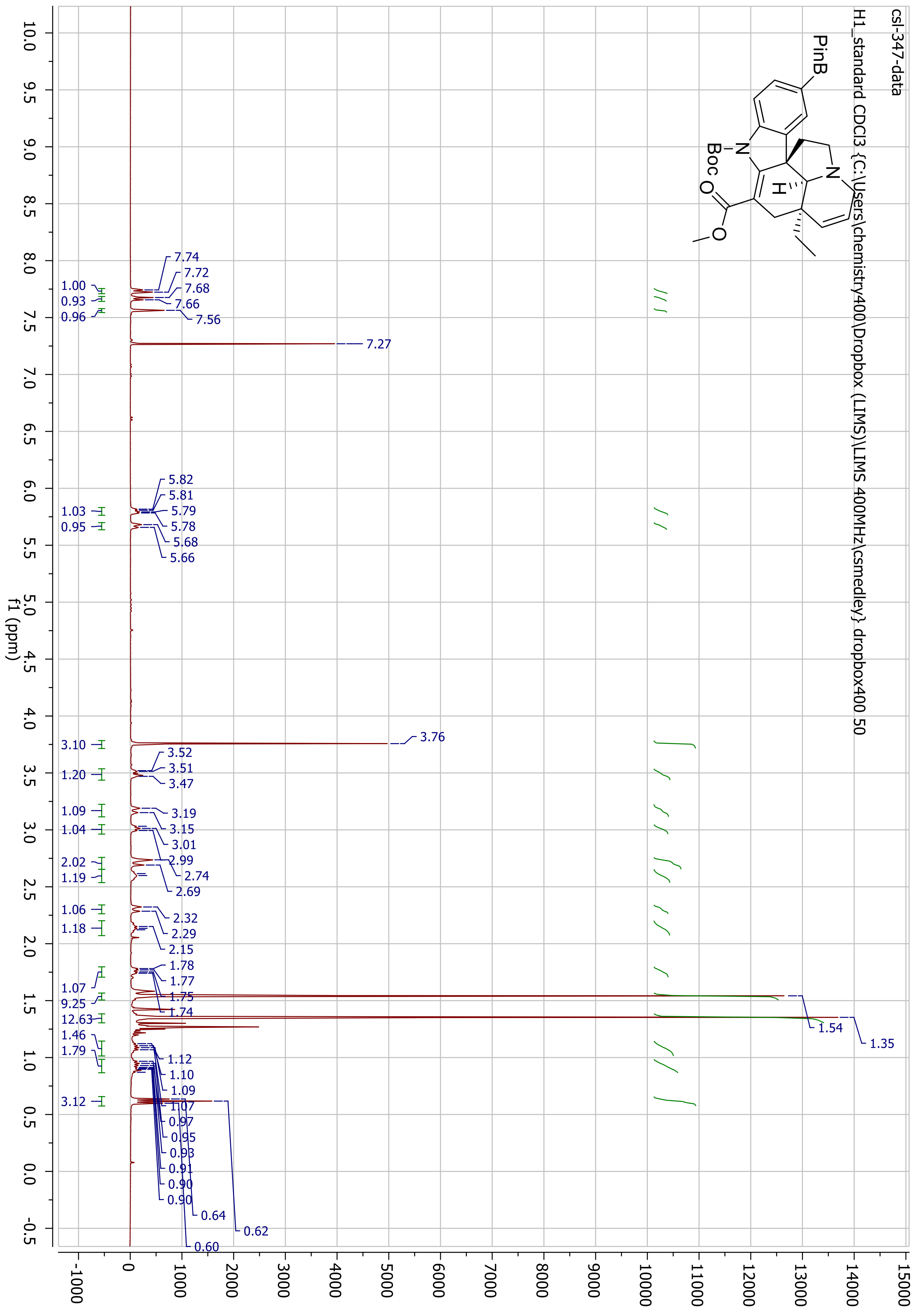

N-Boc-tabersonine-15-boronic acid pinacol ester ^{13}C NMR (13)

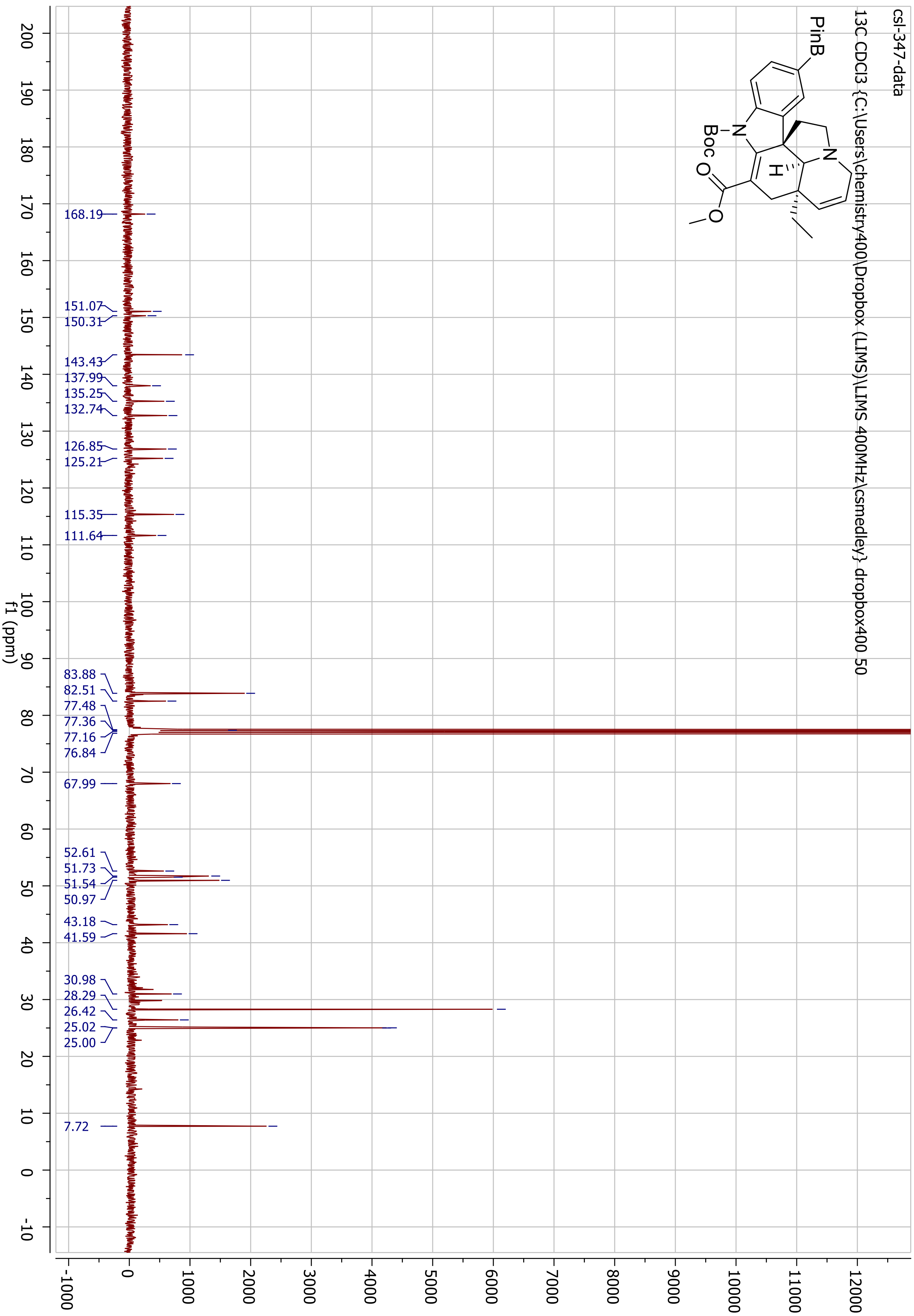

N-Boc-15-hydroxy-tabersonine ^{1}H NMR (14)

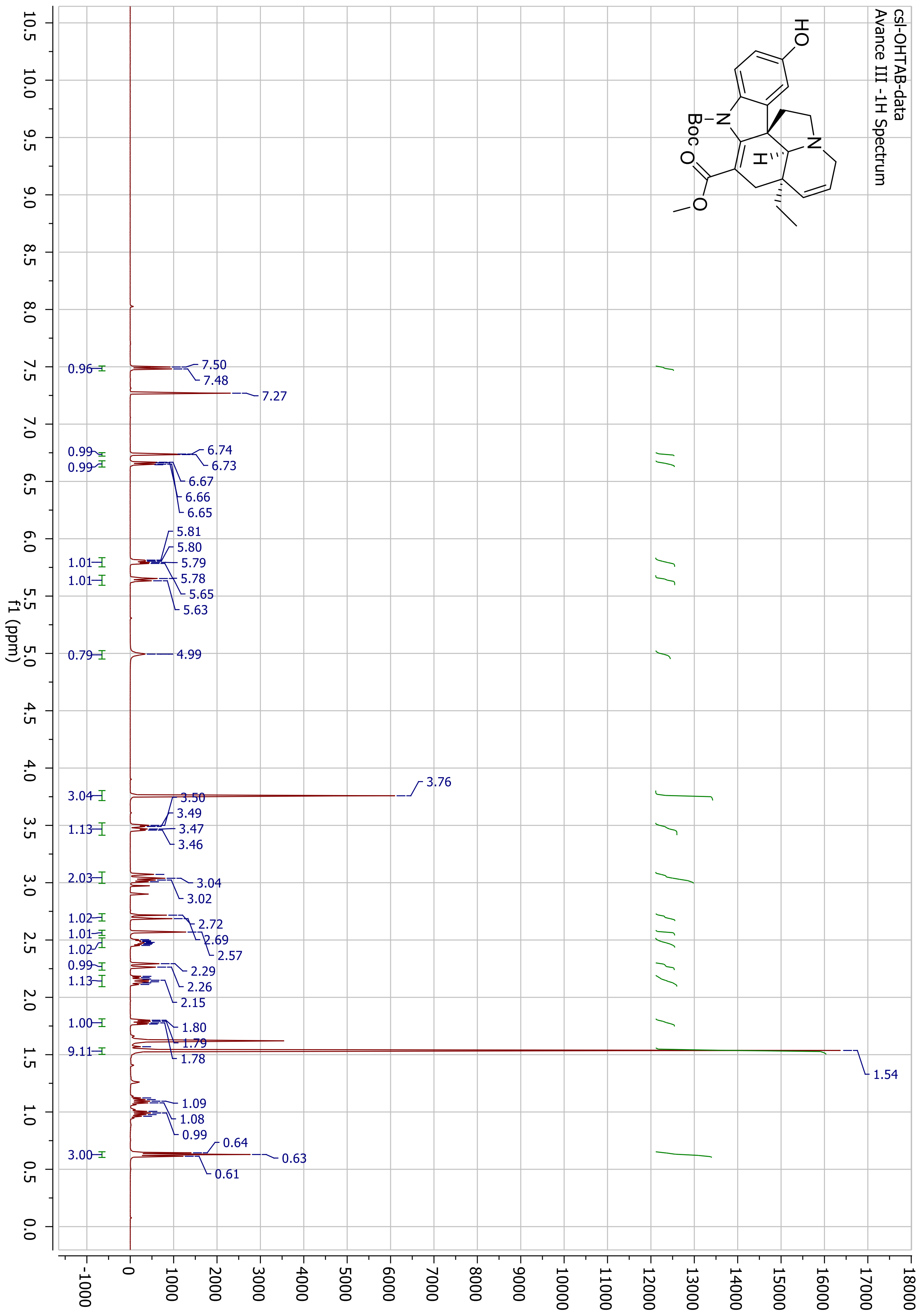

N-Boc-15-hydroxy-tabersonine ^{13}C NMR (14)

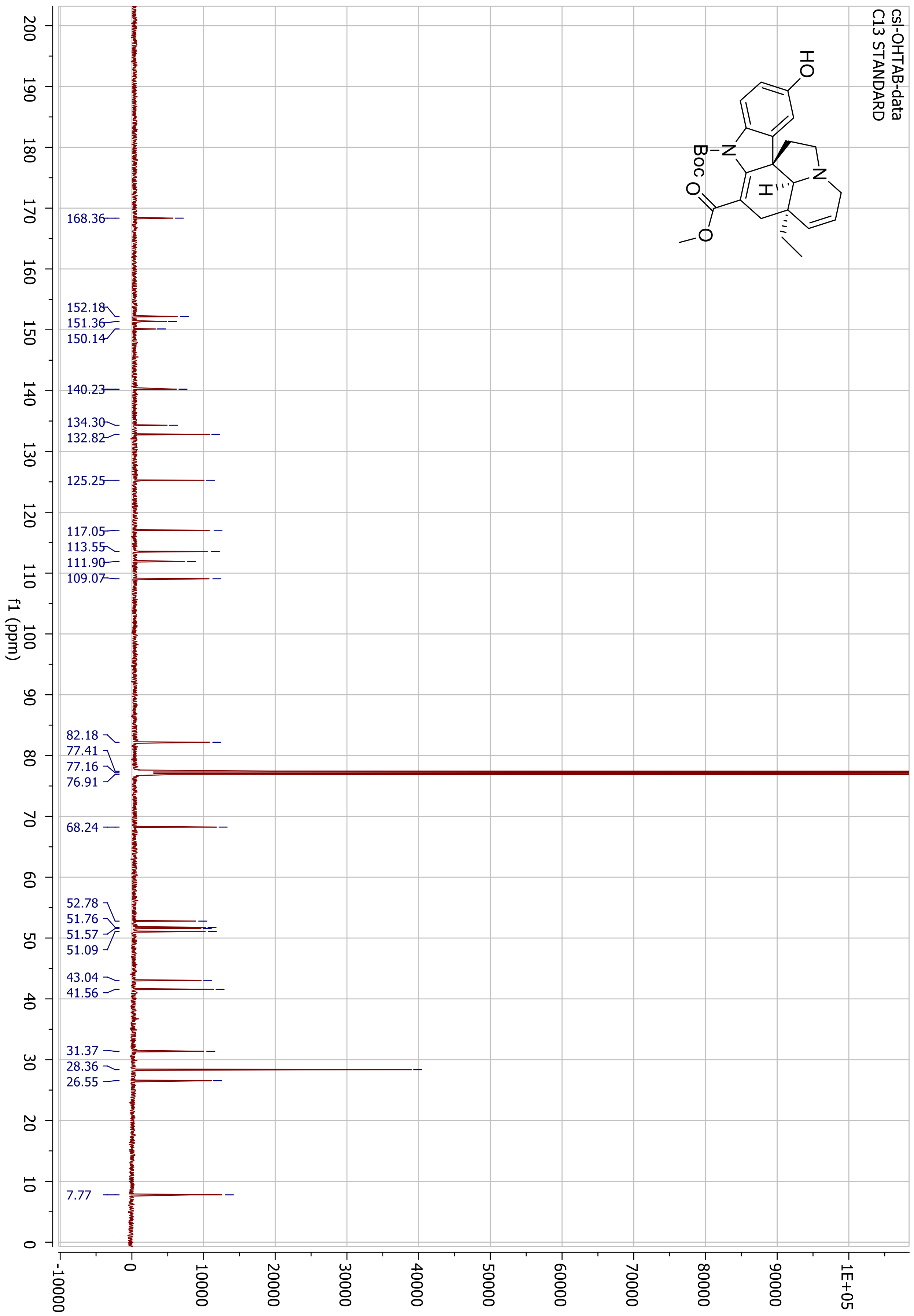

N-Boc-15-hydroxy-16-formyl-tabersonine ^{1}H NMR (15)

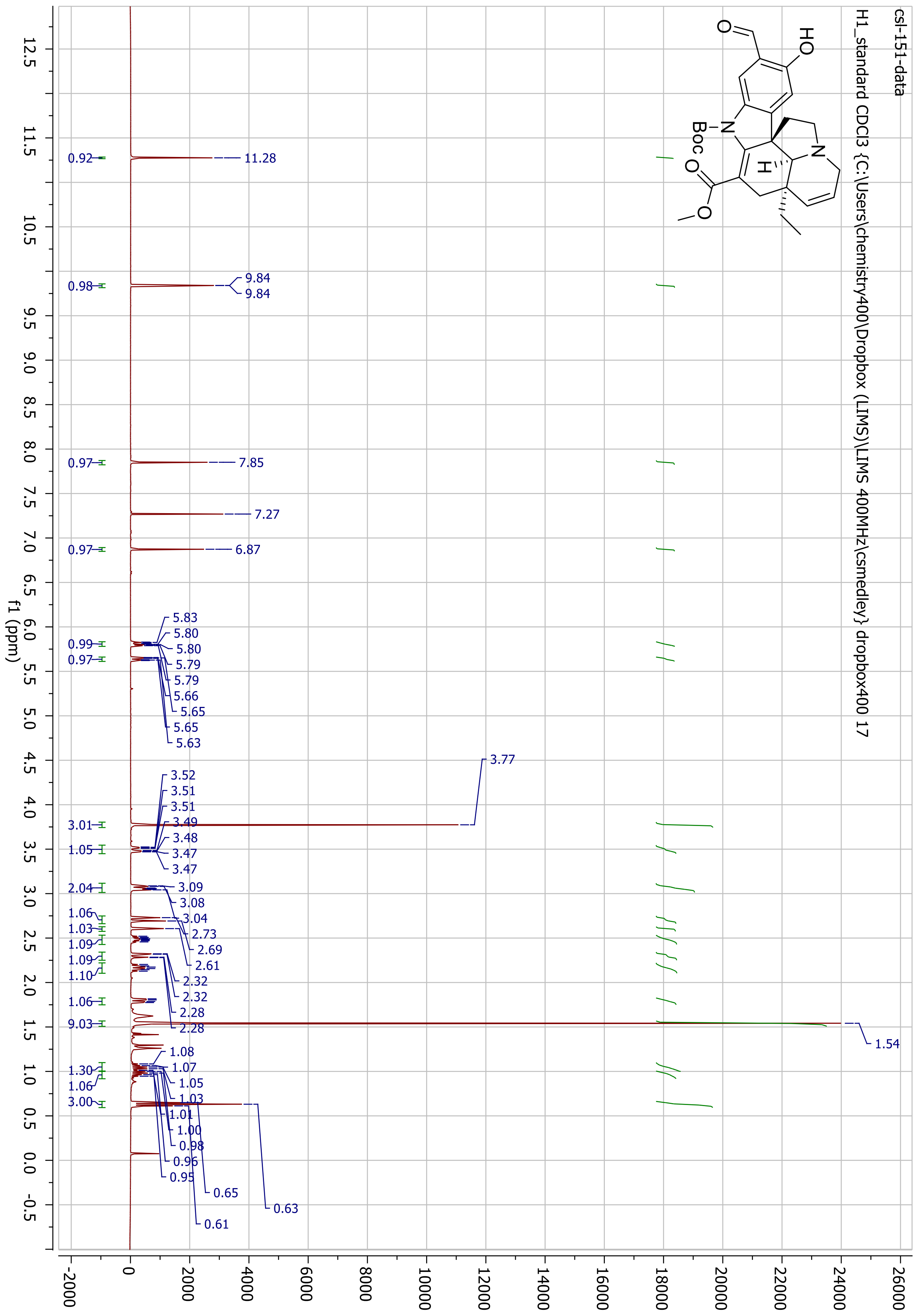

N-Boc-15-hydroxy-16-formyl-tabersonine ^{13}C NMR (15)

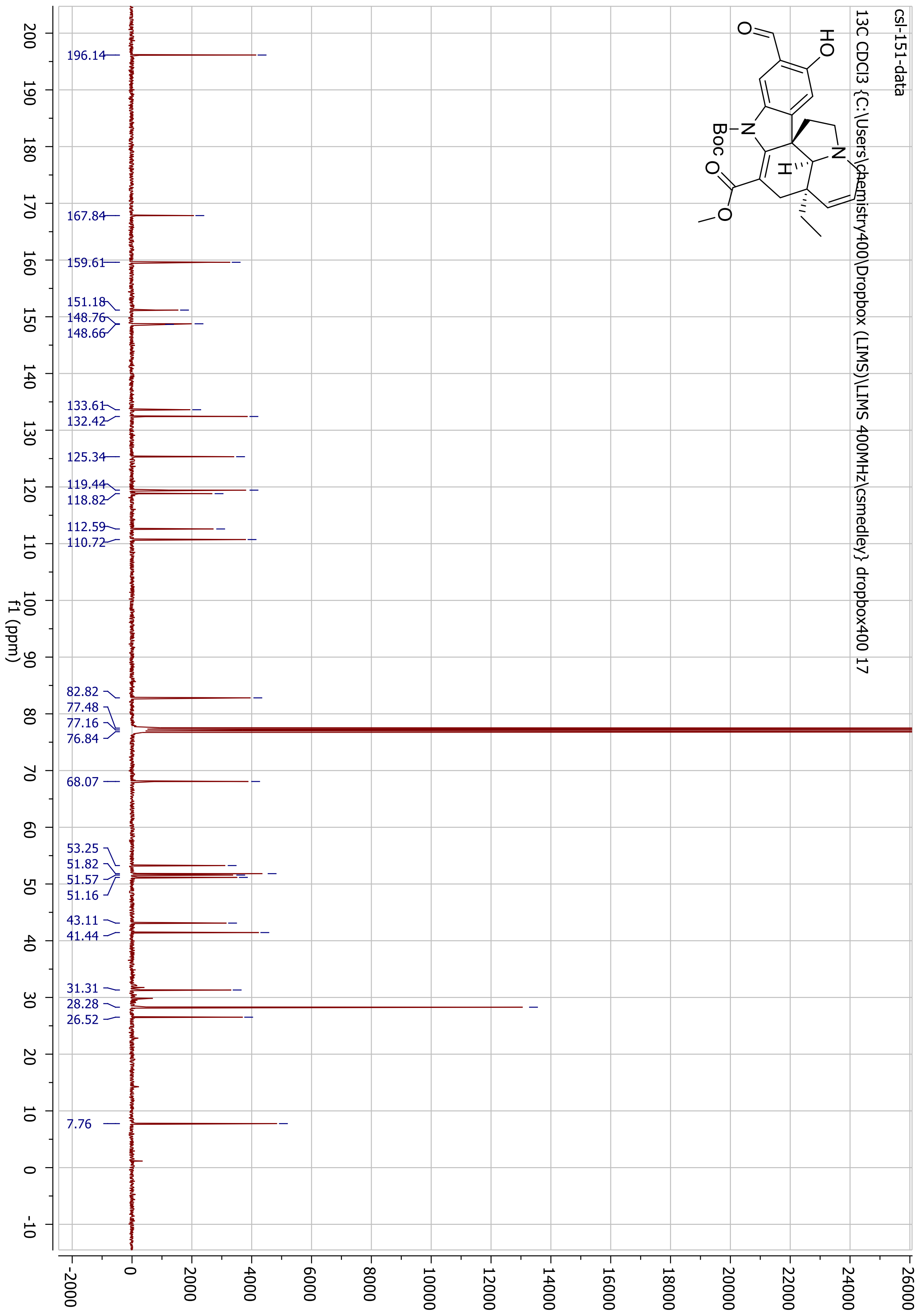

15,16-Dimethoxy-tabersonine ¹H NMR (16)

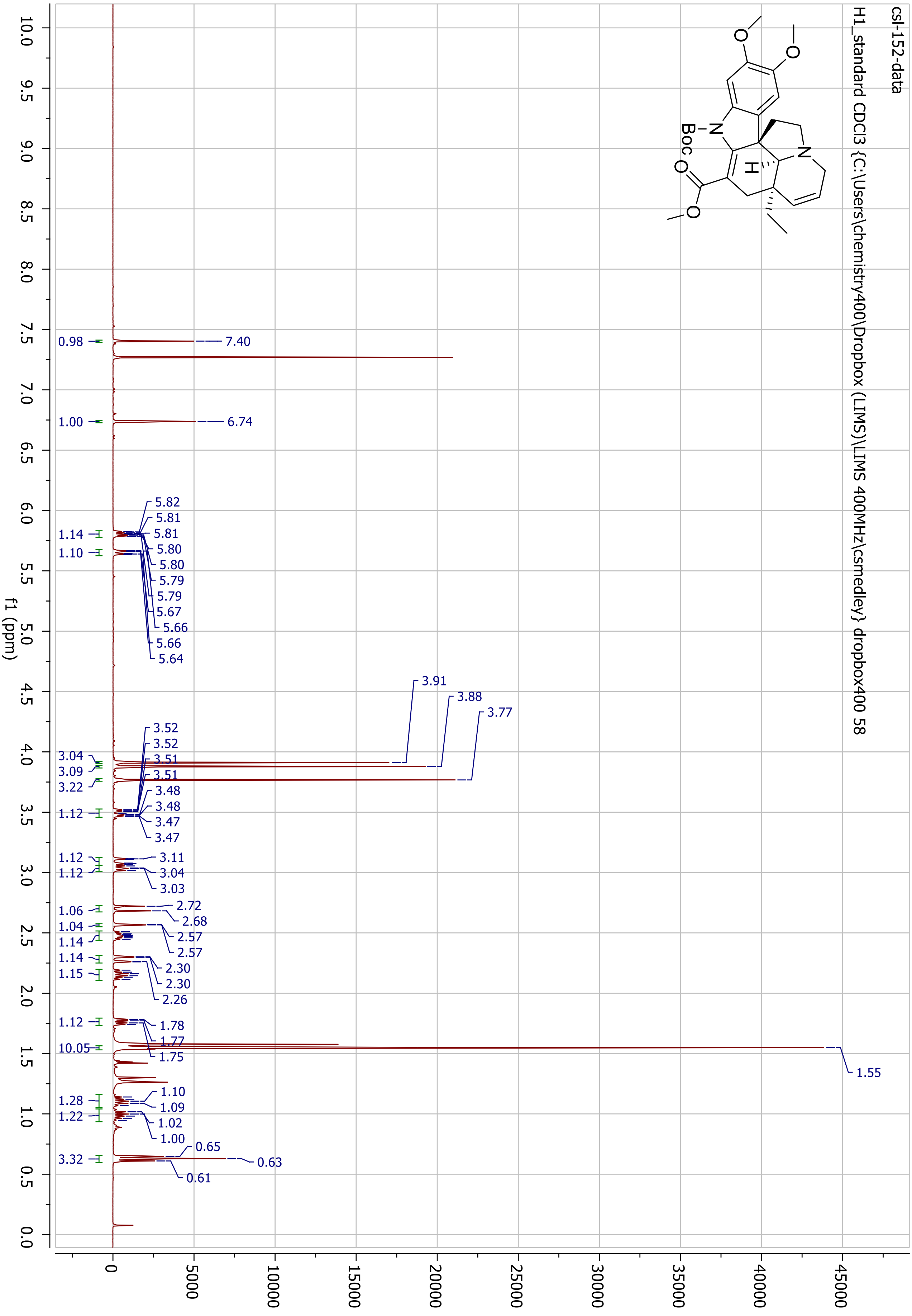

15,16-Dimethoxy-tabersonine ^{13}C NMR (16)

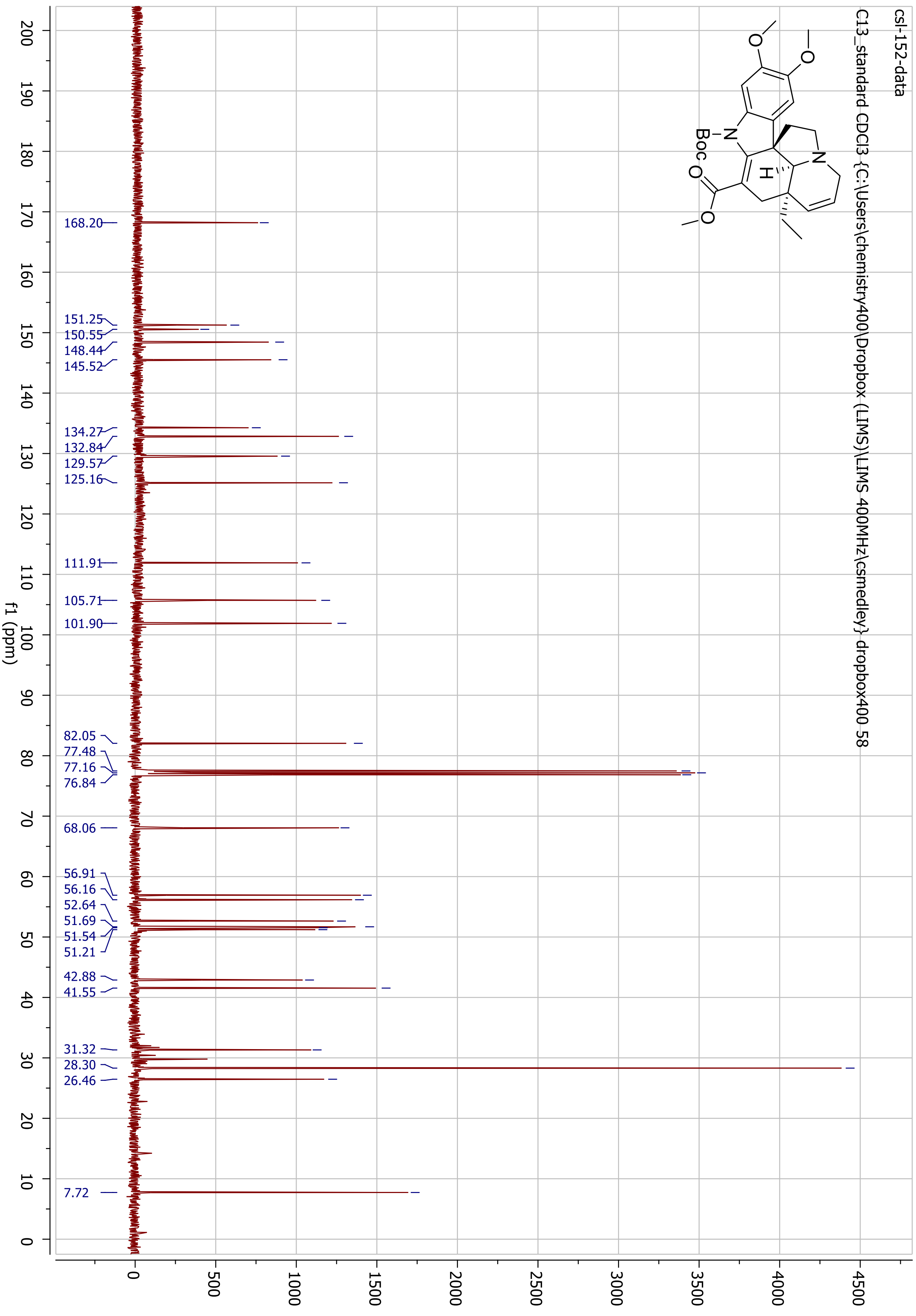

Jerantinine A ^{1}H NMR (1)

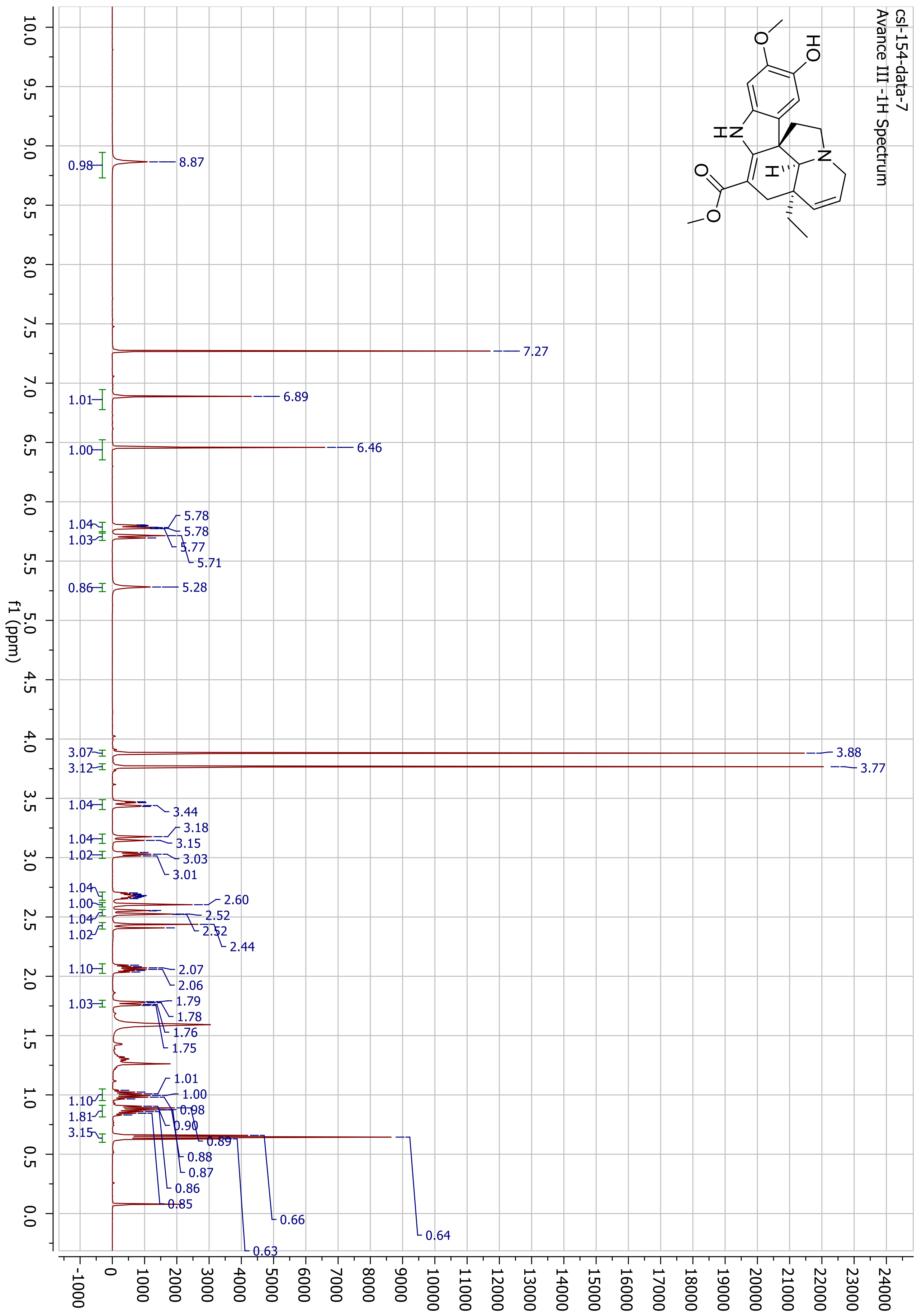

Jerantinine A ^{13}C NMR (1)

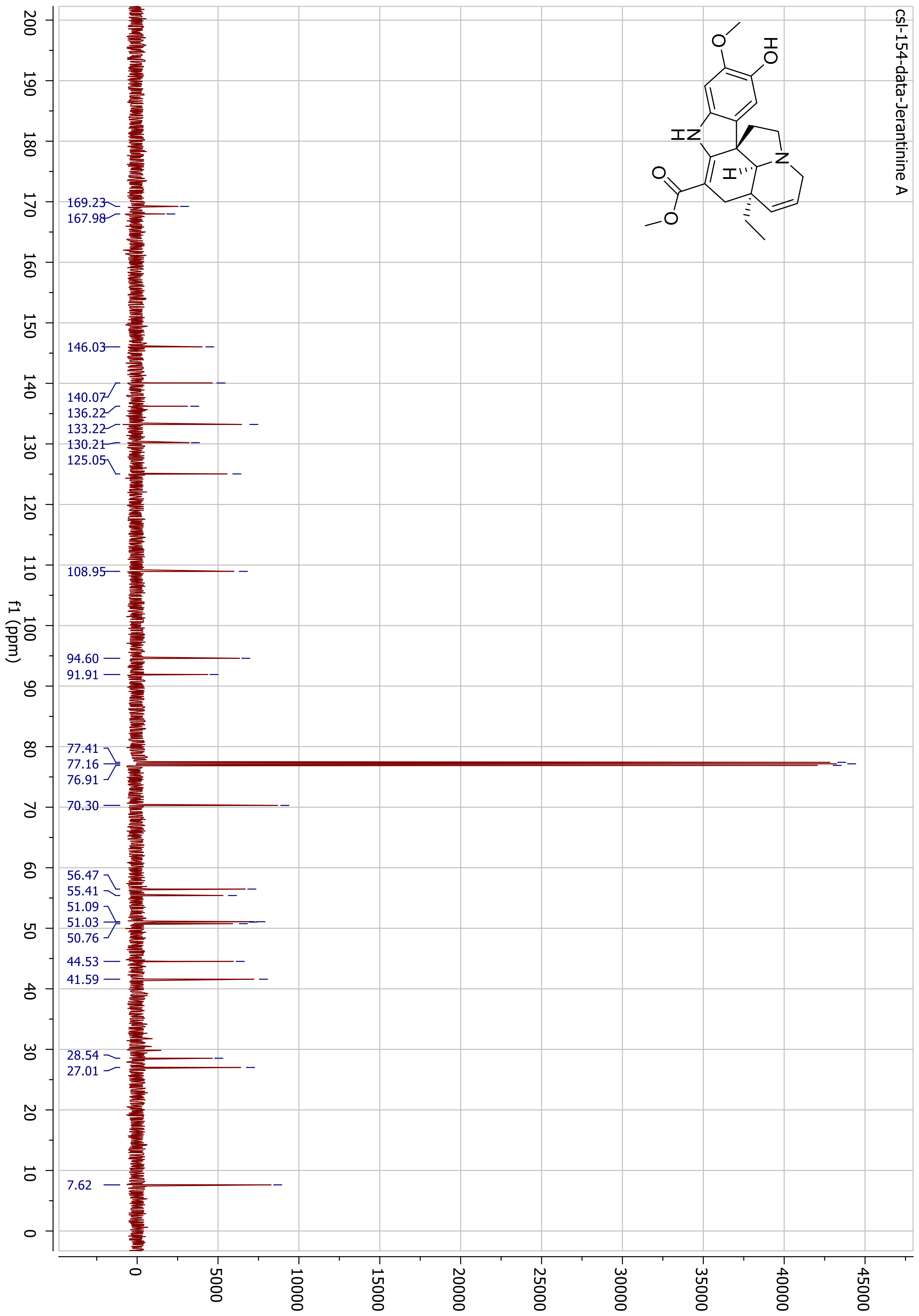

Jerantinine E ^{1}H NMR (3)

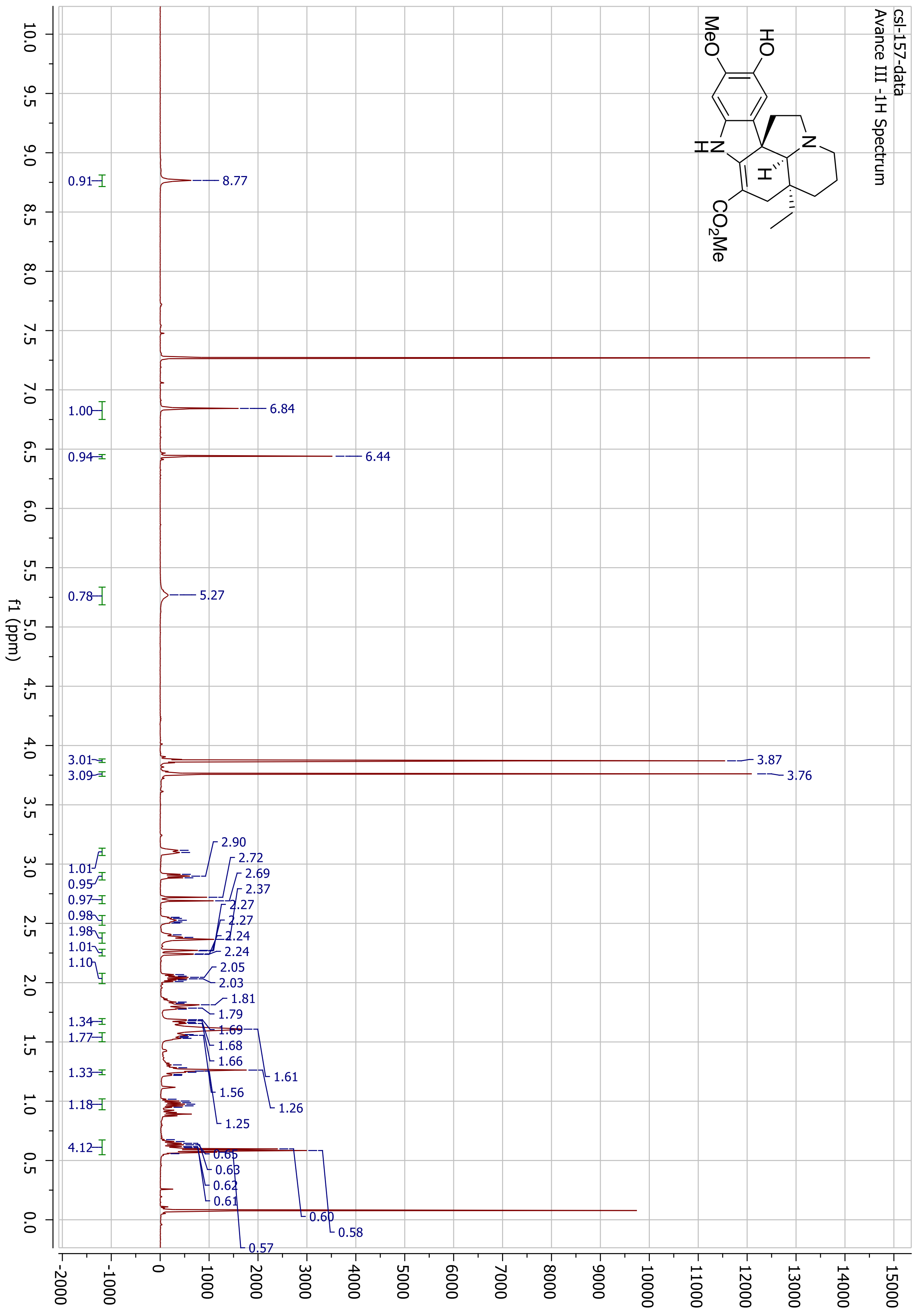

Jerantinine E ^{13}C NMR (3)

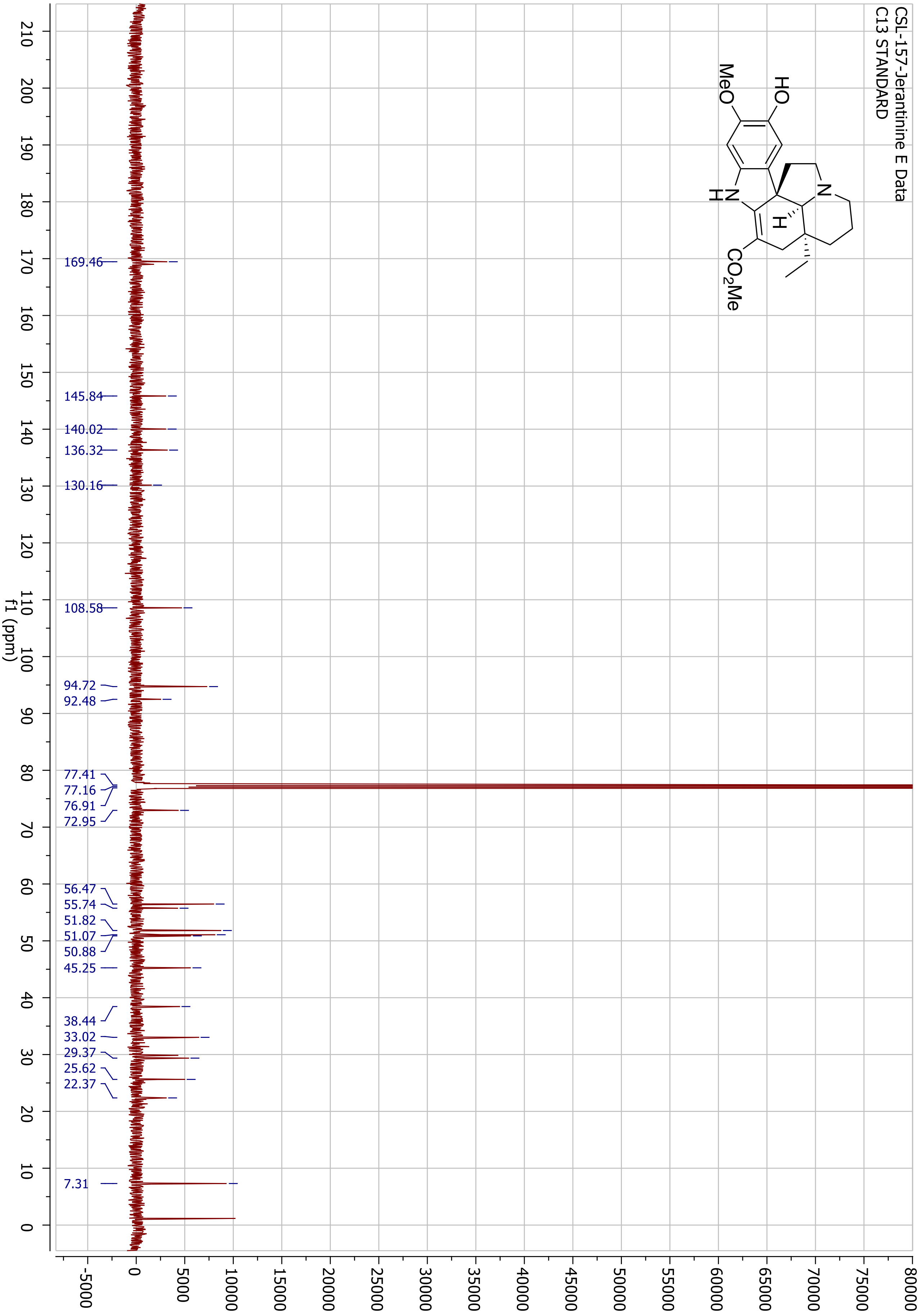

Jerantinine A acetate ^{1}H NMR (7)

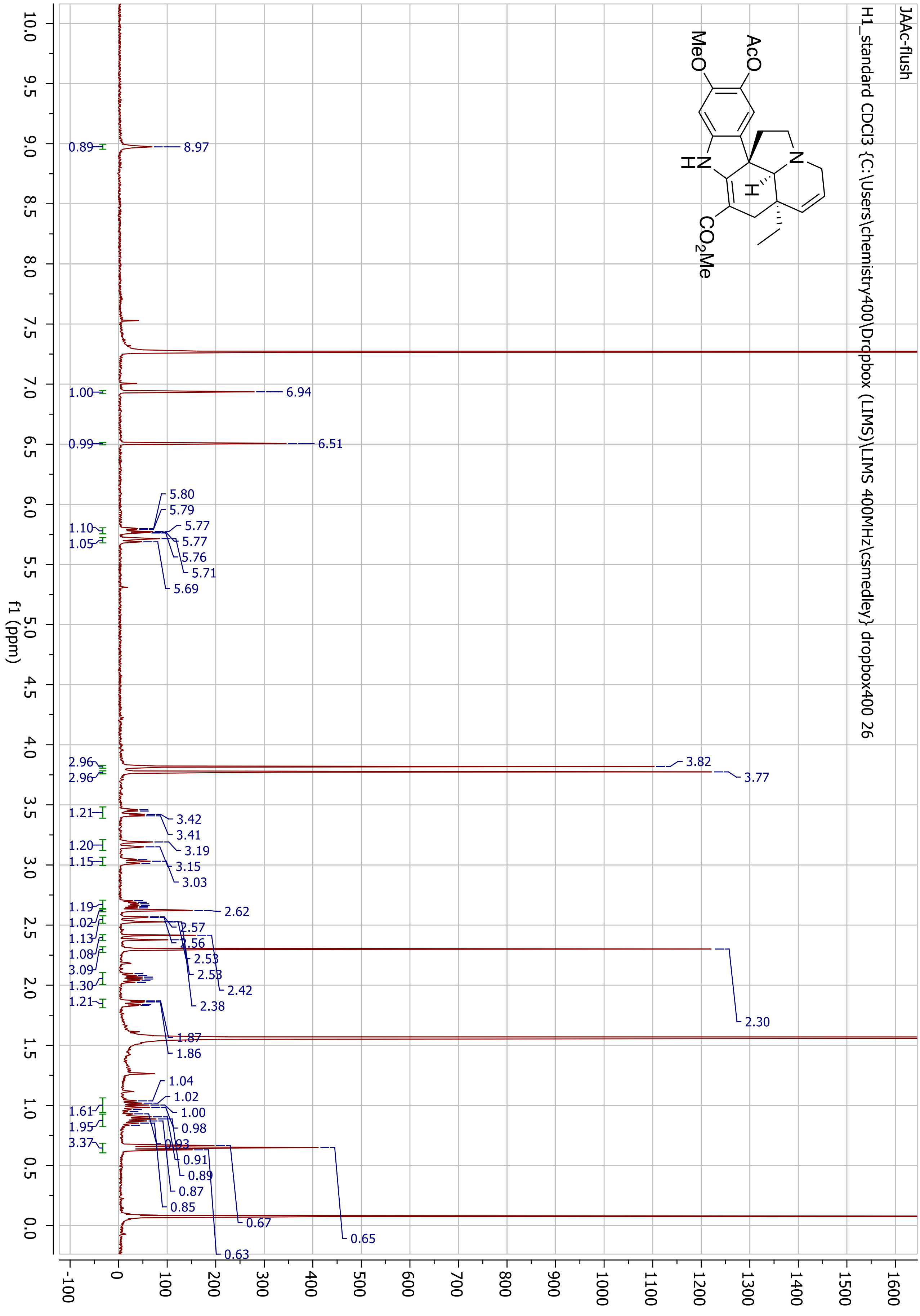

Jerantinine A acetate ^{13}C NMR (7)

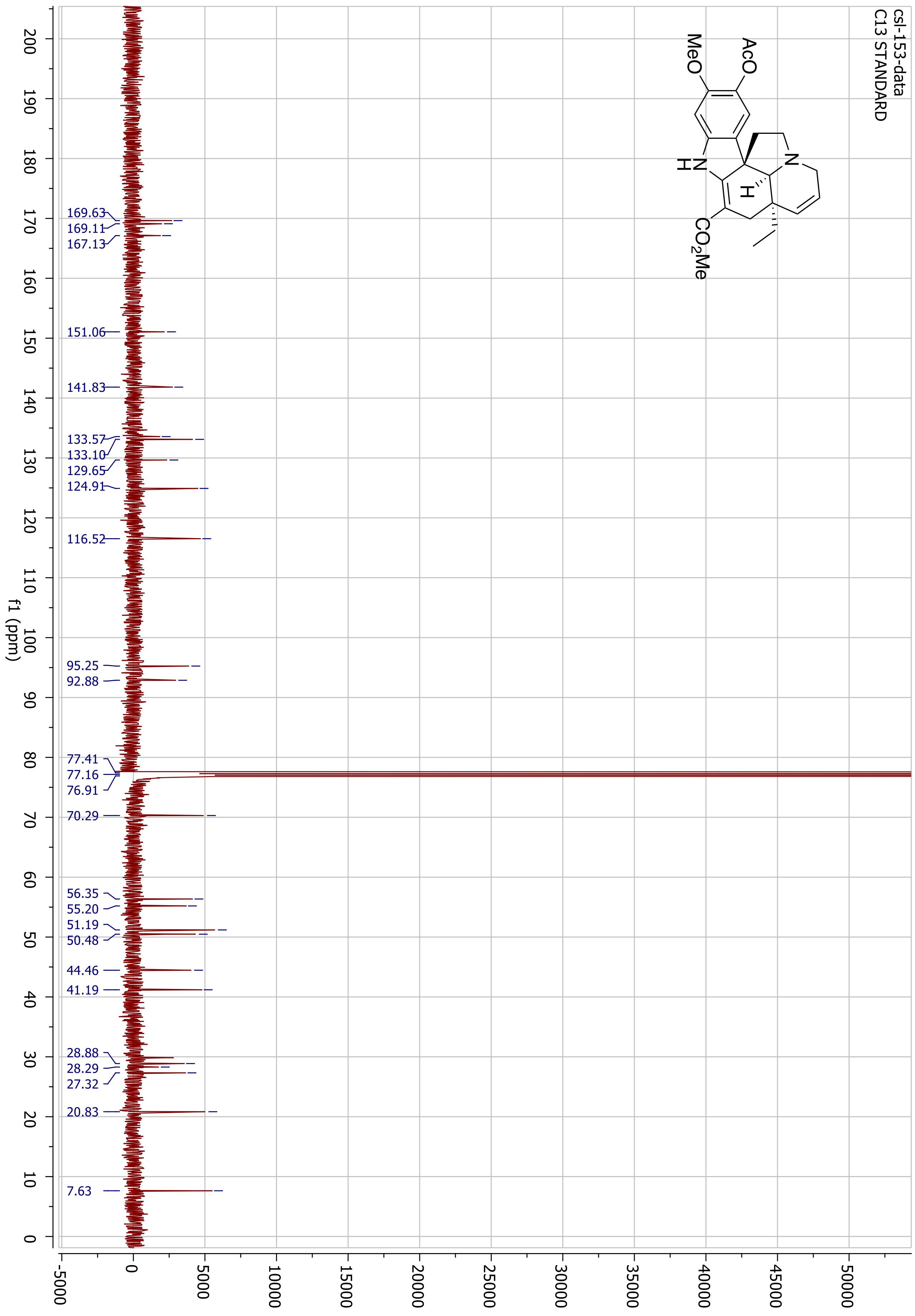

Supplement: Supplementary file 1 — Supplementary Information [file 41598_2018_28880_MOESM1_ESM.pdf]
